# Estimated hourly electricity demand profiles for each county in the contiguous United States

**DOI:** 10.1038/s41597-026-07693-3

**Published:** 2026-07-21

**Authors:** Kodi Obika, Wesley Cole, Marie Rivers

**Affiliations:** National Laboratory of the Rockies, Golden, Colorado USA

## Abstract

In this data descriptor, we present hourly electricity demand estimates for each county in the contiguous United States from 2016 to 2023. The demand profiles represent the sum of two components for each county. The first is the weighted average of reported hourly demand profiles for North American Electric Reliability Corporation balancing authority (BA) regions and subregions, scaled to match annual estimates of county-level retail sales and direct use of electricity and weighted by the estimated percentage of county load served by each BA region or subregion. The second is the weighted average of modeled hourly, county- and sector-level distributed photovoltaic (DPV) capacity factor profiles, scaled to match annual estimates of on-site consumption of DPV-generated electricity for each county and weighted by the percentage of consumption attributable to each sector.

## Background & Summary

Hourly electricity demand is a basis for understanding the electricity grid. It is used as an input to many grid modeling efforts and is integral to evaluating the potential for new grid resources. Time-synchronous wind, solar, and electricity demand profiles are key for planning grid systems that include any amount of wind, solar, and energy storage. Electricity demand is also critical for understanding the value and potential of demand-side resources.

Grid models at the National Laboratory of the Rockies have, for many years, relied on time-synchronous wind, solar, and electricity demand datasets that span the 2007–2013 weather years. These years were chosen because they align with the nationwide wind data available in the WIND Toolkit^1^ for the contiguous United States. However, research has shown that, for planning electricity systems, consideration of a wider range of weather years is desirable^2–4^. Therefore, we create hourly electricity demand profiles spanning years 2016 to 2023 that can be used in conjunction with wind and solar datasets for that same time period. We choose these years because, at the time of writing, they align with the hourly electricity demand data available in the U.S. Energy Information Administration (EIA) Hourly Electric Grid Monitor, which serves as the basis of our dataset. Previous demand datasets used by the National Laboratory of the Rockies were developed at the spatial resolution of the regions used in corresponding grid models, but because finer spatial resolution is becoming increasingly important^5,6^, we develop these demand profiles for each county in the contiguous United States.

Although the demand dataset described here is created with an eye toward electricity grid models, and especially grid planning, we expect the datasets to be valuable for many other use cases, such as evaluating the impacts new technologies might have on demand profiles or calculating correlations between weather and electricity demand. We also note that while electricity demand is highly relevant to electricity pricing, this dataset does not explicitly include any electricity price information.

## Methods

To create hourly electricity demand (load) profiles for each county, we focus our efforts on two primary outputs. The first output is the annual magnitude of electricity consumption. Electricity consumption is the portion of electricity demand that is met by supply. Specifically, it differs from electricity demand in that consumption represents only electricity delivered to customers while demand represents delivered electricity plus dropped load, or electricity that could not be delivered to customers due to insufficient generation, transmission outages, and other grid failures. The second output is the hourly patterns of electricity demand. We create these profiles for the years 2016 through 2023.

Given the high reliability of the electricity system, annual electricity consumption closely mirrors annual electricity demand. Therefore, in the absence of exact measures of electricity demand, we use electricity consumption estimates as a proxy for annual demand. Electricity consumption can be divided into three components:

- Electricity generated by centralized generation facilities (large-scale power plants) and sold to consumers
- Electricity generated by centralized generation facilities and consumed on-site
- Electricity generated by distributed energy resources (DERs) (e.g., rooftop solar installations) and consumed on-site
  Regarding consumption of DER-generated electricity, we are aware only of datasets estimating consumption of electricity produced by distributed photovoltaic (DPV) systems, so we only consider consumption of DPV-generated electricity. Our dataset is therefore intended to represent all electricity consumption in the contiguous United States except that of electricity produced by non-DPV DERs. For brevity, we refer to these three components of electricity consumption as retail sales, direct use, and DPV consumption, respectively, throughout this article.
  We construct county-level hourly load profiles by creating two sets of hourly profiles for each county and adding them: one representing retail sales and direct use and the other representing DPV consumption.
  To create county-level hourly retail sales and direct use profiles, we do the following:
- Estimate annual retail sales for each county.
- Calculate annual direct use for each county.
- Collect reported hourly load and load forecast profiles for North American Electric Reliability Corporation (NERC) balancing authority (BA) regions and subregions. For brevity, we refer to BA regions and subregions as BAs and sub-BAs, respectively, throughout this article.
- Adjust the BA/sub-BA load and load forecast profiles by imputing anomalous values.
- Further adjust the BA/sub-BA load profiles by adding load during periods of reported load loss (electricity demand that could not be met by supply), using the load forecasts to estimate the amount of load lost.
- Estimate each BA/sub-BA’s contribution to each county’s annual retail sales and direct use.
- For each county, scale the BA/sub-BA load profiles so that the profiles’ annual totals equal the BA/sub-BAs’ contributions to the county’s annual retail sales and direct use and add the scaled load profiles.
  To create county-level hourly DPV consumption profiles, we do the following:
- Collect hourly residential and non-residential DPV capacity factor profiles for each county.
- Estimate annual county- and sector-level DPV consumption.
- For each county, scale the sectoral capacity factor profiles so that the profiles’ annual totals equal the county’s annual sectoral DPV consumption values and add the scaled profiles.

**Fig. 1:**
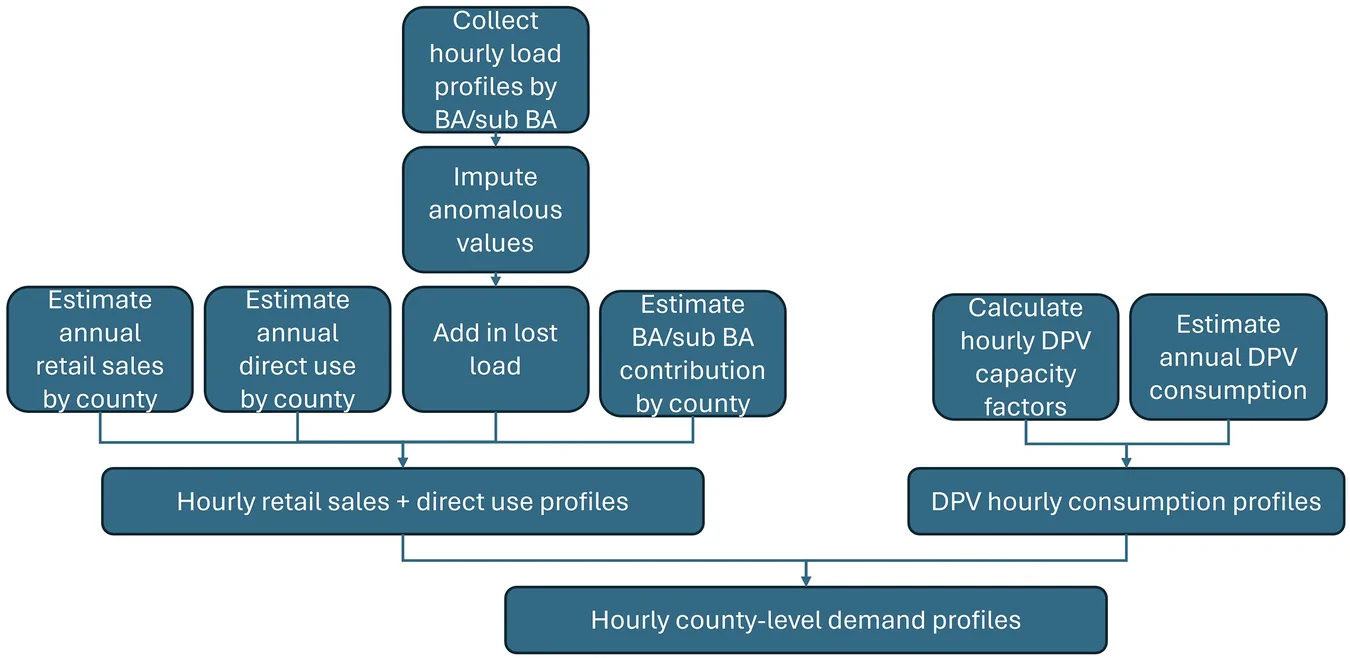
Summary of the process used for creating the hourly county-level demand profiles.

This process is summarized in Fig. 1.

In terms of annual magnitude, the county-level load profiles created here capture only electricity consumption, but adding load to the BA/sub-BA load profiles during periods of reported load loss partially addresses the gap between consumption and demand with respect to the hourly patterns of load. We add load before scaling the BA/sub-BA load profiles to avoid overestimating annual county-level demand. In the end, the county-level hourly load profiles reflect consumption in their annual magnitudes and reflect demand in their hourly patterns. Specific details of these methods are provided in the following sections.

### Estimating annual retail sales for each county

We estimate annual county-level retail sales using state-level electricity sales and county populations. We obtain annual electricity sales labeled by state, utility, and utility ownership type (e.g., investor-owned, cooperative, and behind-the-meter) from the EIA Annual Electric Power Industry Report (EIA-861) (https://www.eia.gov/electricity/data/eia861/) Sales to Ultimate Customers dataset. We obtain annual county populations from the U.S. Census Bureau’s “County Population Totals” tables (https://www.census.gov/data/tables/time-series/demo/popest/2010s-counties-total.html, https://www.census.gov/data/tables/time-series/demo/popest/2020s-counties-total.html).

We calculate annual retail sales for each state by subtracting from the state’s total electricity sales the sales with the “behind-the-meter” utility ownership type, which represent sales from third-party owners of net-metered DERs. We estimate each county’s annual retail sales by allocating statewide retail sales to counties according to each county’s share of state population for the given year.

In summary, retail sales for state *s* in year *t*, r_*st*_, is given by

$${{\rm{r}}}_{{st}}={{\rm{a}}}_{{st}}-{{\rm{b}}}_{{st}}$$

where ${a}_{{st}}$ and ${b}_{{st}}$, respectively, are total electricity sales and “behind-the-meter” sales for state *s* in year *t*. Retail sales for county *c* in year *t*, r_*ct*_, is given by

$${{\rm{r}}}_{{ct}}={{\rm{r}}}_{{st}}\ast {{\rm{p}}}_{{\rm{ct}}}/{{\rm{p}}}_{{\rm{st}}}$$

where p_ct_ is the population of county *c* in year *t* and p_st_ is the population of state *s* in year *t*.

It should be noted that a county’s actual share of statewide retail sales of electricity depends on many factors beyond population, some of which are not proxied well by population. These include commercial and industrial activity, electrification levels, and building stock. As a result, allocating retail sales based solely on share of population is error-prone, as will be shown in the “Technical Validation” section below, and represents a limitation of this dataset. We explored other allocation methods while creating this dataset (also discussed in “Technical Validation”) but found that they did not significantly outperform population-based allocation, so we opted for population-based allocation for the sake of simplicity. Note that our exploration of other methods is by no means comprehensive and other methods more sophisticated than the ones we attempted could perform well enough to justify added complexity.

### Calculating annual direct use for each county

We calculate annual county-level direct use using power plant-level gross generation and on-site consumption figures. We obtain annual gross generation and on-site consumption for all U.S. power plants with capacity greater than 1 megawatt from the EIA Power Plant Operations Report (EIA-923) (https://www.eia.gov/electricity/data/eia923/) Schedules 6 and 7 (“Annual Source and Disposition of Electricity for Non-Utility Generators”). We obtain the location of each power plant from the EIA Annual Electric Power Industry Report (EIA-860) (https://www.eia.gov/electricity/data/eia860/) Schedule 2 (“Plant”). Note that EIA-923 refers to on-site consumption as “direct use”, diverging from our definition of direct use. The former represents the consumption of both self-generated and purchased electricity while the latter represents only consumption of self-generated electricity. We ignore consumption of purchased electricity because it is already accounted for in retail sales estimates.

We calculate each power plant’s annual direct use by taking the minimum of its gross generation and on-site consumption. We calculate each county’s annual direct use by calculating the sum of the direct use values of the power plants located in the county for the given year.

In summary, direct use for power plant *p* in year *t*, d_pt_, is given by

$${{\rm{d}}}_{{\rm{pt}}}=\min ({{\rm{g}}}_{{\rm{pt}}},\,{{\rm{u}}}_{{\rm{pt}}})$$

where g_pt_ and u_pt_, respectively, are the gross generation and on-site consumption for power plant *p* in year *t*. Direct use for county *c* in year *t*, d_*ct*_, is given by

$${{\rm{d}}}_{{ct}}=\sum p\in {\rm{P}}c({{\rm{d}}}_{{\rm{pt}}})$$

where P_*c*_ is the set of power plants located in county *c*.

### Collecting BA and sub-BA load and load forecast profiles

**Fig. 2:**
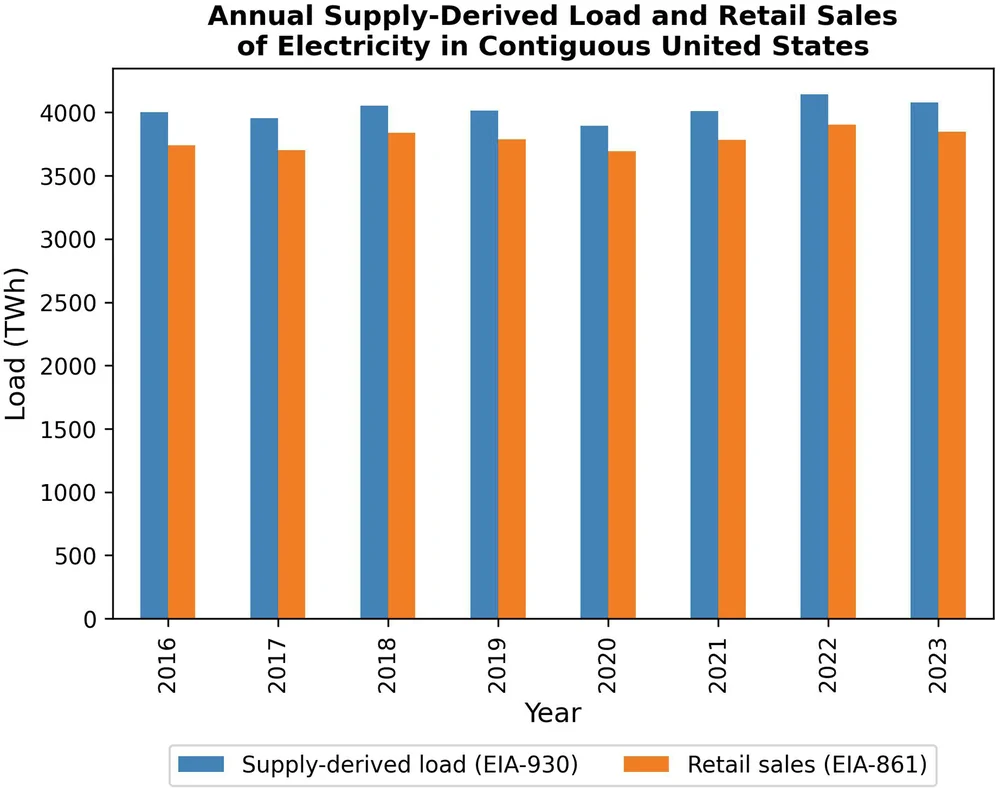
Comparison of supply-derived load and retail sales for the contiguous U.S. Supply-derived load is consistently larger than retail sales because it represents retail sales plus direct use, electricity losses during transmission and distribution, and unaccounted-for electricity.

We obtain hourly load and day-ahead load forecast profiles for years 2016 to 2023 for each NERC BA from the EIA Hourly Electric Grid Monitor (EIA-930) (https://www.eia.gov/electricity/gridmonitor). Load is calculated by taking the difference between metered net generation and metered net interchange for the high-voltage bulk electric power grid, suggesting the load profiles reflect the sum of retail sales, direct use, electricity losses during transmission and distribution, and unaccounted-for electricity (the portion of electricity supply that cannot be attributed to identified consumption or losses). At times throughout this article, we refer to this load as “supply-derived load” to distinguish it from other types of load being discussed. Figure 2 illustrates the annual differences between supply-derived load and retail sales for the contiguous U.S. The hourly profiles are published in Coordinated Universal Time (UTC) using the hour ending time reporting convention, meaning a load value corresponding to timestamp 15:00 (hour ending 15) represents the time-weighted average load from 14:00 up to and excluding 15:00.

For some BAs, EIA-930 provides hourly load profiles for subregions of the BA, or sub-BAs, which the EIA-930 documentation defines as “a further level of geographic disaggregation (for example, load zones, weather zones, operating company areas, local BAs, etc.) within a BA’s electric system.” For most of the BAs that report subregional data, the documentation also provides links to maps of the sub-BAs, which we use to determine the entities that the sub-BAs represent. Where maps are not provided, we infer sub-BAs’ corresponding entities based on their naming and publicly available online information. Specifically, maps are not provided for the California Independent System Operator (CISO) and Southwest Power Pool (SWPP) BAs. CISO’s sub-BAs share their names with major utilities operating in the BA, such as Pacific Gas & Electric and Southern California Edison, so we assume CISO’s sub-BAs correspond to the BA’s operating company areas. SWPP’s sub-BAs share their names with the BA’s legacy balancing authorities, such as Nebraska Public Power District and Southwestern Power Administration (https://www.spp.org/documents/58198/2017%20spp%20lole%20study%20report.pdf#page=14), so we assume SWPP’s sub-BAs correspond to these local legacy BAs.

The first year in which EIA-930 provides comprehensive load data for most sub-BAs is 2019, so we collect hourly pre-2019 load profiles and day-ahead load forecast profiles for each sub-BA from their respective BA’s website or application programming interface (API) where available. Table 1 outlines, for each applicable BA’s set of sub-BAs, the entities corresponding to the sub-BAs, the availability of external data (load and load forecast data outside of EIA-930), and the time zone and time reporting convention external data are published in. In the table, each BA’s name is followed by its corresponding EIA-930 identifier and its most widely recognized abbreviation (if different from the EIA-930 identifier). Where necessary, we modify external data to be in UTC and to use the hour ending convention for consistency with the data from EIA-930. Note we could not find any data online for the sub-BAs of the Public Service Company of New Mexico (PNM) BA, so we only use its BA-wide load and load forecast profiles in the processes described in subsequent sections. In subsequent sections, we refer to the seven BAs with comprehensive sub-BA load profiles as “parent BAs.”

**Table 1:** Entities corresponding to applicable BA’s sub-BAs and the availability, time zone, and time reporting convention of sub-BA data outside of EIA-930.

BA	Entities Corresponding to Sub-BAs	External Sub-BA Load Profiles?	External Sub-BA Day-Ahead Load Forecast Profiles?	Time Zone of External Sub-BA Profiles	Time Reporting Convention of External Sub-BA Profiles
California Independent System Operator (CISO/CAISO)	CAISO operating company areas	Yes	Yes	UTC	Hour ending
Electric Reliability Council of Texas, Inc. (ERCO/ERCOT)	1	Yes	Yes, starting on June 30, 2017.	Central Prevailing Time	Hour ending
New England ISO (ISNE/ISONE)	ISONE load zones (https://www.iso-ne.com/about/key-stats/maps-and-diagrams/#load-zones)	Yes	Yes	Eastern Prevailing Time	Hour ending
Midcontinent Independent System Operator, Inc. (MISO)	MISO local resource zones (https://etariff.ferc.gov/TariffSectionDetails.aspx?tid=1162&sid=272842)	Yes	Yes, starting on Aug. 30, 2022.	Eastern Standard Time	Hour ending
New York Independent System Operator (NYIS/NYISO)	New York Control Area load zones (https://www.nyiso.com/documents/20142/1397960/nyca_zonemaps.pdf)	Yes	Yes	Eastern Prevailing Time	Hour ending
PJM Interconnection, LLC (PJM)	PJM transmission zones (https://www.pjm.com/library/-/media/B7455E69D97B45FFB390EEFAD84AD50D.ashx)	Yes	Yes, for nine out of 20 sub-BAs.	UTC	Hour beginning
Public Service Company of New Mexico (PNM)	N/A	No	No	N/A	N/A
Southwest Power Pool (SWPP/SPP)	SPP legacy balancing authorities	Yes	No	UTC	Hour ending

The process for acquiring sub-BA data outside of EIA-930 is outlined below:

- CAISO sub-BA load and load forecast profiles are downloaded via the CAISO Open Access Same-time Information System (OASIS) API.
  - Usage instructions are available at https://www.caiso.com/documents/oasis-interfacespecification_v5_1_2clean_fall2017release.pdf.
  - For load, we use the query name “SLD_FCST” and market run ID “ACTUAL.”
  - For load forecasts, we use the query name “SLD_FCST” and market run ID “DAM.”
  - OASIS is also available as an interactive portal at https://www.caiso.com/systems-applications/portals-applications/open-access-same-time-information-system-oasis.
- ERCOT sub-BA load and load forecast profiles are downloaded from https://www.ercot.com/gridinfo/load/load_hist and https://data.ercot.com/data-product-archive/NP3-565-CD, respectively.
  - Note that creating an account is required to access the load forecasts.
  - Load forecast files are posted every thirty minutes, with each file containing hourly forecasts for the current and next seven days using a variety of forecast models. To extract one set of day-ahead forecasts, we download only the files posted at 14:30 on a given day, which is when ERCOT runs its day-ahead reliability unit commitment process each day (https://www.ercot.com/files/docs/2010/02/12/ruc_overview__wind.pdf#page = 11). From those files, we select only the rows for which the “InUseFlag” is specified as “Y”, which indicates that ERCOT used the forecast at the time of publication, and for which the time indices correspond to the day after the day of posting.
- MISO sub-BA load and load forecast profiles are downloaded via the MISO Data Exchange Load, Generation, and Interchange API.
  - Instructions for Data Exchange registration, which is required to access the API, are available at https://cdn.misoenergy.org/MISO%20Data%20Exchange%20User%20Guide674561.pdf?v=20250124142839.
  - Usage instructions for the API are available at https://data-exchange.misoenergy.org/api-details#api=load-generation-and-interchange-api.
  - We use the “get-v1-real-time-date-demand-load-state-estimator” operation for load. See https://data-exchange.misoenergy.org/api-details#api=load-generation-and-interchange-api&operation=get-v1-real-time-date-demand-load-state-estimator.
  - We use the “get-v1-forecast-date-load” operation for load forecasts. See https://data-exchange.misoenergy.org/api-details#api=load-generation-and-interchange-api&operation=get-v1-forecast-date-load. Note that the operation provides day-ahead forecasts by default, i.e., if no “init” parameter is specified in the API call.
- ISONE sub-BA load and load forecast profiles are downloaded from https://www.iso-ne.com/isoexpress/web/reports/load-and-demand/-/tree/zone-info.
  - Both load and load forecasts are contained in the “SMD Hourly Data” files, under the columns “RT_Demand” and “DA_Demand,” respectively.
- NYISO sub-BA load and load forecast profiles are downloaded from the “Custom Reports” interactive tool on the BA’s website at https://www.nyiso.com/custom-reports.
  - For load, we use the “Load Data—Integrated Real-Time Actual” report.
  - For load forecasts, we use the “Load Data—Load Forecast” report.
- PJM sub-BA load and load forecast profiles are downloaded from https://dataminer2.pjm.com/feed/hrl_load_metered and https://dataminer2.pjm.com/feed/load_frcstd_hist, respectively.
  - Multiple load forecasts are posted for a given operating hour. To extract a single day-ahead forecast for each operating hour, we select the row with the latest time of evaluation that occurred before 11 AM Eastern time on the day before the operating hour, which is when PJM’s day-ahead market bid period closes (https://www.pjm.com/-/media/DotCom/committees-groups/committees/mic/2023/20230412/20230412-item-02-3–real-time-values-manual-11-revisions–redline.ashx).
- SPP sub-BA load profiles are downloaded from https://portal.spp.org/pages/hourly-load.

We generally obtain hourly load profiles for years 2019 to 2023 for each sub-BA from EIA-930, but in some cases, the sub-BA load profiles provided by EIA-930 contain clear and substantial errors (e.g., the 2020 load profile for PJM’s Rockland Electric (East) zone sub-BA, or RECO, contains only one unique load value) or have large portions of missing data. In these cases, we replace load values with the data available outside of EIA-930. These cases are listed below.

- The 2016–2023 profiles for the RECO sub-BA are taken from the PJM website.
- The 2019 profiles for all ERCO sub-BAs are taken from the ERCOT website.
- The January 2019 subset of the Pennsylvania Power & Light zone (PL) sub-BA load profile is taken from the PJM website.
- The subset of the Pacific Gas and Electric (PGAE) sub-BA profiles spanning Jan. 1, 2019, 00:00 Coordinated Universal Time (UTC) to May 2, 2019, 07:00 UTC is taken from the CAISO OASIS API.
- The subset of the Valley Electric Association (VEA) sub-BA profiles spanning Dec. 23, 2019, 09:00 UTC to May 2, 2020, 07:00 UTC is taken from the CAISO OASIS API.

### Adjusting BA and sub-BA load and load forecast profiles

**Fig. 3:**
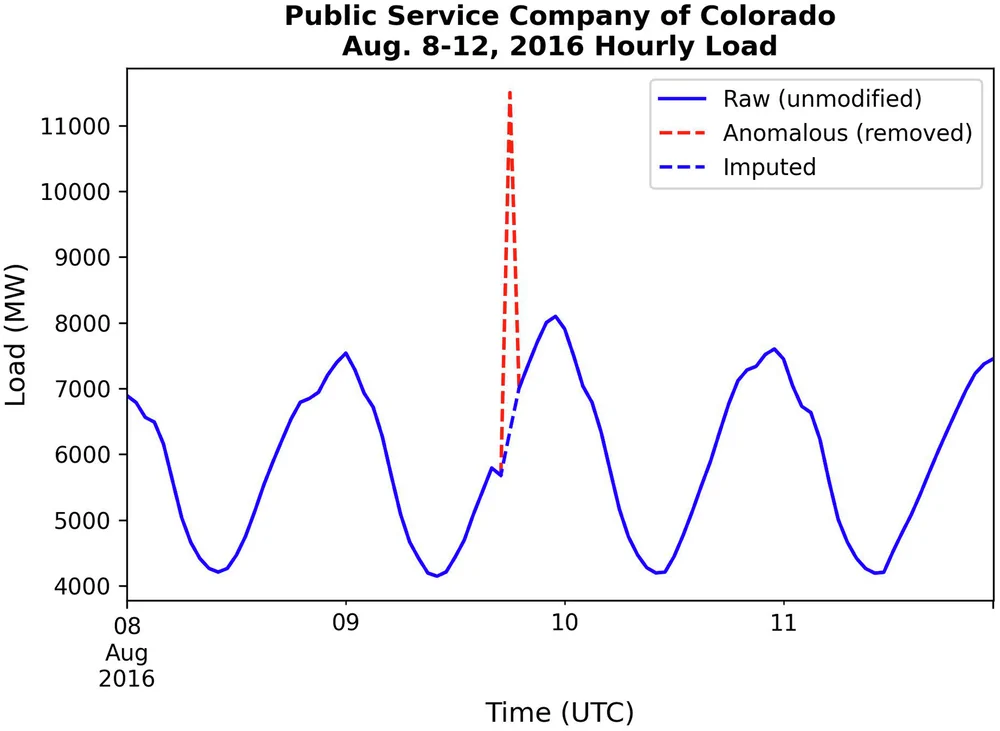
Aug. 8-12, 2016 hourly load for PSCO before and after imputation.

The hourly load and load forecast profiles initially have some missing or otherwise anomalous values. To address these issues, we use a method developed by Ruggles *et al*.^7^ that screens hourly load data for anomalies and fills in or replaces these values. This is done by applying a multiple imputation by chained equations (MICE) technique that predicts load values for a given BA/sub-BA using linear regressions on the BA/sub-BA’s load values during the hour immediately before and after anomalous hours, the BA/sub-BA’s average load during the same seasons of year and hours of day as the anomalous hours, and the load values of other BA/sub-BAs during the anomalous hours. As an example, Fig. 3 shows the effect of the method on a portion of the load profile for the Public Service Company of Colorado (PSCO) BA that initially contained an anomalous load value. The load profiles of two of the BAs, Ohio Valley Electric Corporation and Seminole Electric Cooperative, contain too many anomalies for the MICE technique to be effective, so we do not incorporate those profiles in the processes described in subsequent sections. We apply the MICE technique to the load and load forecast profiles of all other BA/sub-BAs, resulting in the adjustment of 1.5% of raw values overall.

The hourly load profiles also reflect instances of load loss. We attempt to replace these periods’ reported load values with approximations of what load would have been without these losses. To do this, we obtain the start and end times, affected areas (states and/or counties), reporting entities, and disturbance types (e.g., load shedding, electric service loss) for the major load loss events that occurred from 2016 to 2023 from the EIA Electric Power Monthly (EPM) (https://www.eia.gov/electricity/monthly/) Table B.2 (“Major Disturbances and Unusual Occurrences, Prior Year”), which we refer to as EPM Table B.2. Note that we obtain the table for a given year from the following year’s March issue of EPM.

We use the reporting entity and affected area of each event to determine which load profiles were impacted by the event. If the reporting entity is a BA, we assume the BA’s load profile was impacted by the event. If the reporting entity is a utility, we assume the load profiles of the BAs the utility operated in in the given year were impacted by the event. The Sales to Ultimate Customers dataset assigns a BA code to most electricity sales, so we determine the BAs that each utility operated in by selecting the set of BAs associated with the sales made by the utility. If we find the event to be associated with any parent BAs, we determine the event’s impacted sub-BAs in one of two ways. If the reporting entity shares its name or alias with a sub-BA (e.g., Nebraska Public Power District, which is the name of SWPP’s NPPD sub-BA), we associate the event with that sub-BA. Otherwise, we associate the event with the sub-BAs whose boundaries contain the event’s affected area. To determine this, we obtain county boundaries from the U.S. Census Bureau’s 2022 TIGER/Line County shapefile (https://www2.census.gov/geo/tiger/TIGER2022/COUNTY/), BA boundaries from the EIA Balancing Authorities shapefile (https://atlas.eia.gov/datasets/eia::balancing-authorities) and sub-BA boundaries from the EIA RTO Regions shapefile (https://atlas.eia.gov/maps/eia::rto-regions). In some cases, we modify the given BA/sub-BA boundaries to be more consistent with official maps or to address clear errors. These cases are outlined in the following section, titled “BA and sub-BA boundary modifications.”

We use the start and end time of each event to determine which portions of the applicable load profiles were impacted. EPM Table B.2 does not specify the time zones the start and end times are reported in, but based on inspection of a few events and online reports concerning these events, we deduce that entities report event times in their local time zones, with the reported times reflecting daylight saving time when applicable. Table 2 lists the reporting entity, disturbance type, start time, and end time of each of these events, as reported in EPM Table B.2, and the corresponding time zone of each event, as identified online. We obtain the local time zone of each BA from EIA-930. We derive the local time zone of each utility by determining the time zone with which the utility’s service territory shares the most area. To do this, we obtain utility service territory boundaries from the EIA Electric Retail Service Territories shapefile (https://atlas.eia.gov/datasets/geoplatform::electric-retail-service-territories) and time zone boundaries from the U.S. Bureau of Transportation Statistics Time Zones shapefile (https://geodata.bts.gov/datasets/usdot::time-zones) and calculate their areas of intersection for each reporting utility. For each event in Table 2, we also specify the reporting entity type (BA or utility) and the reporting entity’s local time zone, as specified in EIA-930 (for BAs) or as determined by the spatial intersections between utility service territory and time zone boundaries (for utilities). For each event in EPM Table B.2 with defined start and end times, we determine the impacted portions of the applicable load profiles by converting the start and end times to UTC and determining their corresponding hour endings. We ignore events with undefined start or end times.

**Table 2:** Example entries from EPM Table B.2 and supplemental time zone information.

Reporting Entity	Disturbance Type	Event Start Time	Event End Time	Event Time Zone Reported Online	Reporting Entity Type	Reporting Entity Local Time Zone
Cooperative Energy	Public appeal to reduce electricity use	02/17/2021 5:00 PM	02/17/2021 10:00 PM	Central Standard Time (https://cooperativeenergy.com/cooperative-energy-miso-ask-members-to-limit-electricity-use-2/)	Utility	Central
MISO	Load shedding	02/15/2021 5:40 AM	02/16/2021 1:11 PM	Eastern Standard Time (https://cdn.misoenergy.org/2021%20Arctic%20Event%20Report554429.pdf#page=6)	BA	Eastern
Pacific Gas & Electric Co.	Load shedding	08/14/2020 6:36 PM	08/14/2020 8:42 PM	Pacific Daylight Time (https://www.kpp.energy/pagename/category/news/page/187/#:~:text=operator%20on%20Aug-,.,-14%20and%20Aug)	Utility	Pacific

For the BA/sub-BAs and time periods for which we have load forecast profiles, we apply the following process to replace values impacted by load loss events:

- Create copies of the load and load forecast profiles and filter out load loss periods (hours corresponding to load loss events) from the copies.
- Fit a linear regression model using the filtered load forecast profile as the predictor variable and the filtered load profile as the response variable.
- Apply the regression model to the original (unfiltered) load forecast profile to create a “predicted” load profile.
- Concatenate the original load profile and the portions of the predicted load profile corresponding to load loss events and then drop duplicate hours from the combined profile, keeping the maximum value for each hour.
  It should be noted that combining load and load forecasts is error-prone and can create inconsistencies due to forecast errors (e.g., systematic overestimation or underestimation of actual load). We attempt to correct for these errors through the linear regression method described above (i.e., by using a predicted load profile rather than the load forecasts directly), but we acknowledge that the predicted load profile still represents a modeled approximation of actual load.
  For the sub-BAs and time periods for which we do not have load forecasts, we use the MICE technique to replace values impacted by load loss events by doing the following:
- Replace load values with “NA” during periods of load loss that we do not have load forecasts for.
- Rerun the MICE technique, passing as inputs all the BA/sub-BA load profiles, including all adjustments described so far.
- For each hour of load loss periods that we do not have load forecasts for, compare the new load value (the load value assigned by the MICE technique) to the original load value (the load value before the replacement with “NA”) at that hour. If the original value exceeds the new value, replace the new value with the original value.

**Fig. 4:**
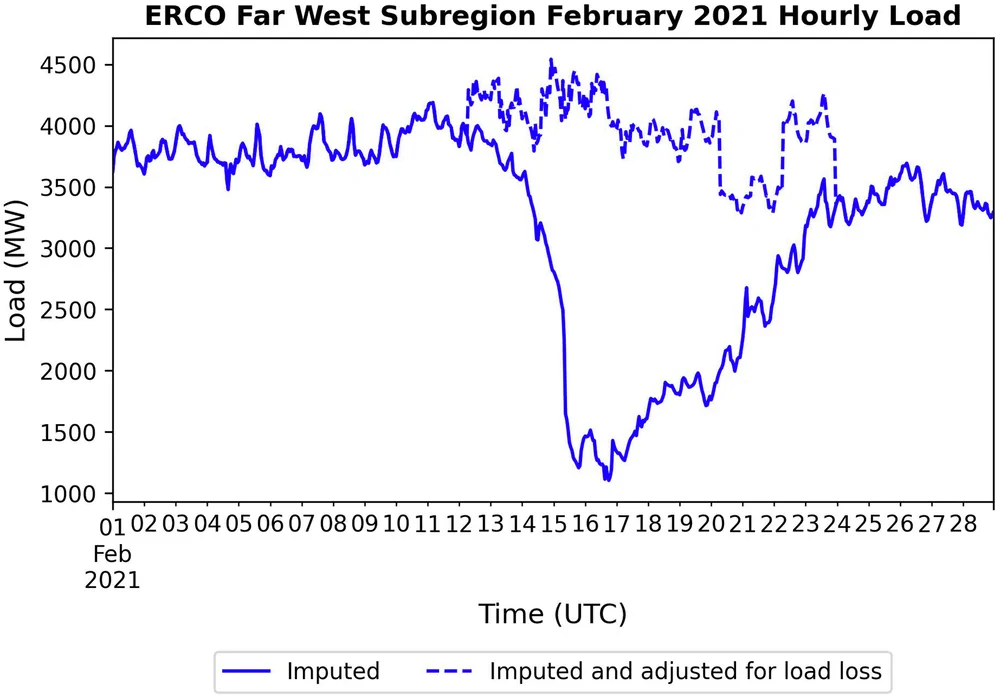
February 2021 hourly load for ERCO Far West before and after load loss adjustment. The large drop in load in the initial load profile reflects the electric service losses and load shedding that occurred in Texas during Winter Storm Uri.

Figure 4 shows the February 2021 load profile for the ERCO Far West (FWES) sub-BA before and after running this method.

In some cases, unexpected peaks in the load profiles are either not corrected by the MICE technique or are created by the load loss adjustment process, so we make additional modifications to correct them. These cases are listed below. It should be noted that these peaks were identified by visual inspection, and therefore there could be similar but less obvious problems that we were unavailable to identify.

- The 2021 profile for the NorthWestern Corporation (NWMT) BA has unexpectedly high load values from May 14 to May 23 that were not identified as anomalous by the MICE technique and that we could not trace to any extreme weather events. To resolve this, we manually replace those values with “NA” before running the MICE technique for the second time as part of the load loss adjustment process and do not replace the values assigned by the MICE technique.
- The MICE step of the load loss adjustment produces an unexpected peak of 36 GW on Feb. 16, 2021, for MISO’s Zones 8, 9, and 10 (8910) sub-BA, which is higher than MISO’s estimate of peak load (without load management) for the sub-BA in February 2021 of 31 GW (see the “Winter 2020/2021 Peak Load Forecasts and Actual Loads for Event Area” figure in the “FERC - NERC - Regional Entity Staff Report: The February 2021 Cold Weather Outages in Texas and the South Central United States”, available at https://www.nerc.com/globalassets/our-work/reports/event-reports/february_2021_cold_weather_report.pdf#page=33; note that MISO South corresponds to the 8910 sub-BA). To resolve this, we replace the sub-BA’s hourly load values on Feb. 16, 2021, with the average of the load values for each hour of Feb. 15, 2021, and Feb. 17, 2021, after performing the load loss adjustment.

### BA and sub-BA boundary modifications

We make the following modifications to the BA and sub-BA boundaries obtained from the EIA Balancing Authorities and RTO Regions shapefiles.

- The initial boundaries for PNM contain a misplaced service area in Oregon. We create new boundaries by taking the spatial difference between the initial boundaries and Oregon.
- The initial boundaries for the Western Area Power Administration (WAPA) Desert Southwest Region (WALC) BA contain service areas in Northern California and Oregon that are not included in the region map published by WAPA (https://www.wapa.gov/about-wapa/regions/). We create new boundaries by calculating the spatial intersection between the initial boundaries and the combined area of Arizona, New Mexico, Utah, California’s San Bernardino, Riverside, and Imperial counties, and Clark County, Nevada.
- The initial boundaries for SWPP’s sub-BAs are generally inconsistent with the transmission zone map published by SPP (https://www.spp.org/documents/58198/2017%20spp%20lole%20study%20report.pdf#page=14). We create new sub-BA boundaries by combining county boundaries, initial sub-BA boundaries, and new boundaries that we draw manually using QGIS 3.44, an open-source geographic information system software, and using the transmission zone map as a reference. Specifically, we follow these steps in order:
  - To create new boundaries for the WAPA Upper Great Plains East (WAUE) sub-BA, we draw the portions of the sub-BA that exist in Nebraska. Then, we calculate the spatial intersection between the SWPP BA boundaries and the combined area of Montana, North Dakota, South Dakota, Minnesota, Iowa, and Wyoming and combine that intersection with the drawn Nebraska service area.
  - To create new boundaries for NPPD, we take the spatial difference between the state of Nebraska and the portion of Nebraska contained in the boundaries of other SWPP sub-BAs.
  - We draw new boundaries for the AEPW American Electric Power West (CSWS), Grand River Dam Authority (GRDA), Oklahoma Gas and Electric Co. (OKGE), Southwestern Public Service Company (SPS), and Western Farmers Electric Cooperative (WFEC) sub-BAs.
  - To create new boundaries for the Westar Energy (WR) sub-BA, we take the spatial difference between the state of Kansas and the portion of Kansas contained in the boundaries of other SWPP sub-BAs.
  - To create new boundaries for the Empire District Electric Company (EDE) sub-BA, we draw new boundaries and take the spatial difference between the drawn boundaries and all other SWPP sub-BAs.
  - After creating the new sub-BA boundaries mentioned above, we calculate the spatial difference between the SWPP BA and the combined area of the sub-BAs (including initial sub-BA boundaries for those not mentioned above). We then fill in unclaimed areas of the BA by determining the counties the areas belong to and assigning each area to the sub-BA its county shares the most area with or is closest to (if the county does not intersect any sub-BAs).
- The initial boundaries for PJM’s sub-BAs have some inconsistencies with the transmission zone map published by PJM (https://www.pjm.com/library/-/media/B7455E69D97B45FFB390EEFAD84AD50D.ashx). We resolve the inconsistencies by combining county boundaries, initial sub-BA boundaries, and utility service territory boundaries. Specifically, we follow these steps in order:
  - To create new boundaries for the Allegheny Power zone (AP) sub-BA, we take the spatial difference between Virginia and the service territory of the Virginia Electric & Power Co utility and then select the portions of that area that intersect with Virginia’s Frederick, Winchester, Clarke, Warren, Page, Rappahannock, Madison, Highland, and Greene counties. We then combine that area with the spatial difference between the sub-BA’s initial boundaries and the portion of the initial boundaries that intersect Virginia.
  - To create new boundaries for the American Electric Power zone (AEP) sub-BA, we combine the sub-BA’s initial boundaries with the Michigan counties of Van Buren, St. Joseph, Berrien, Kalamazoo, and Cass and Virginia’s Norton City and Wise County.
  - To create new boundaries for the Delmarva Power and Light zone (DPL) sub-BA, we combine the sub-BA’s initial boundaries with the Virginia counties of Accomack and Northampton.
  - To create new boundaries for the Dominion Virginia Power zone (DOM) sub-BA, we take the spatial difference between Virginia and all other PJM sub-BA boundaries. We then combine that area with the portion of DOM’s initial boundaries that intersect North Carolina.
  - To create new boundaries for the Potomac Electric Power zone (PEP) sub-BA, we take the spatial difference between the combined area of Maryland’s Calvert, Prince George’s, Howard, Montgomery, St. Mary’s, and Charles counties and all other PJM sub-BA boundaries. We then combine that area with the boundaries of Washington D.C.
  - To create new boundaries for the American Transmission Systems, Inc. zone (ATSI) sub-BA, we take the spatial difference between the combined area of Pennsylvania’s Beaver, Butler, Lawrence, Mercer, Allegheny, and Crawford counties and all other PJM sub-BA boundaries. We then combine that area with ATSI’s initial boundaries.
  - To create new boundaries for the Pennsylvania Power & Light zone (PL) sub-BA, we take the spatial difference between the combined area of Pennsylvania’s Wyoming and Luzerne counties and all other PJM sub-BA boundaries. We then combine that area with PL’s initial boundaries.
  - To create new boundaries for the Atlantic Electric zone (AE) sub-BA, we combine AE’s initial boundaries with Cumberland County, New Jersey.

**Fig. 5:**
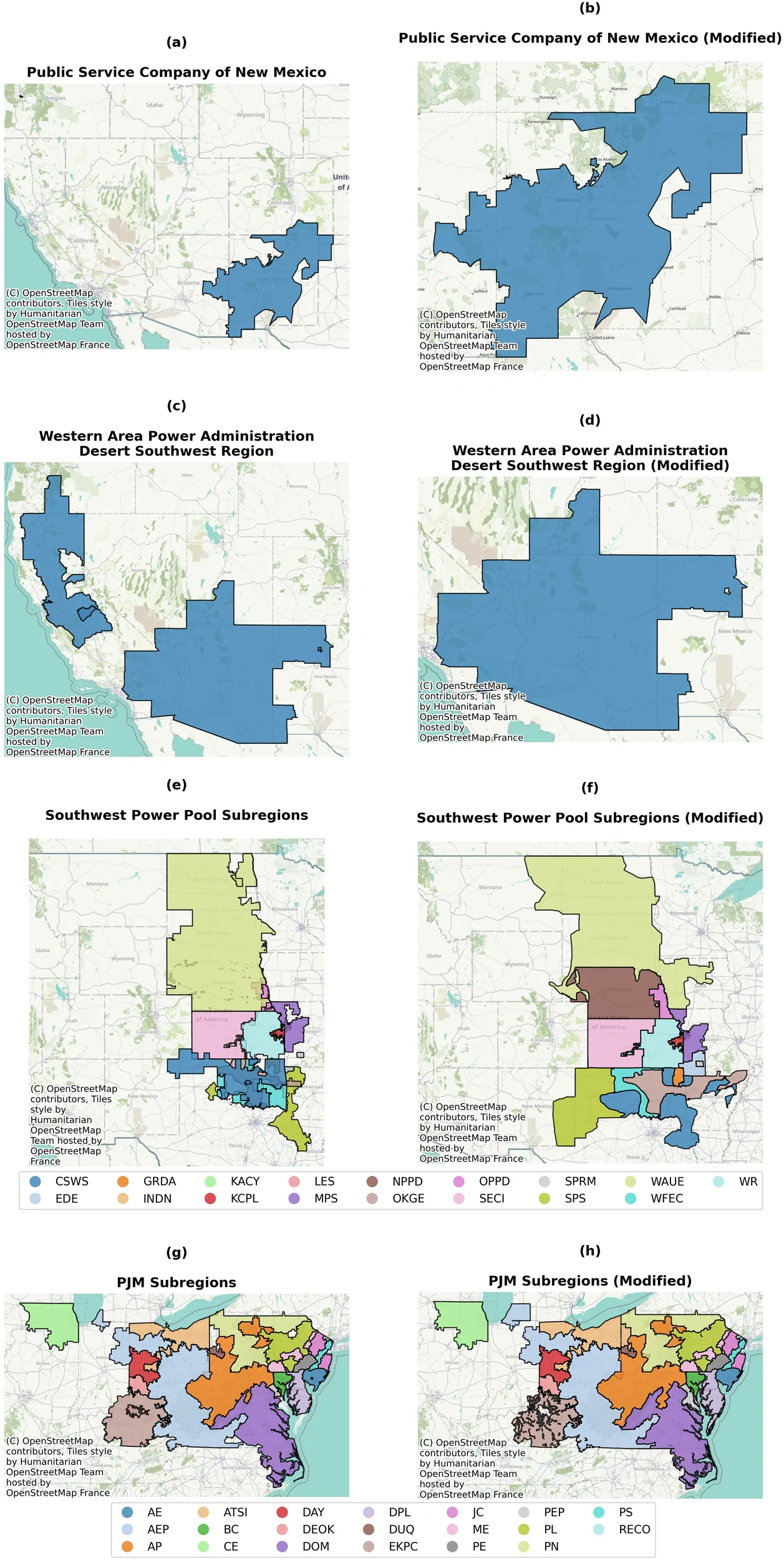
Initial and modified BA/sub-BA boundaries. (**a**) Initial PNM BA boundaries. **(b)** Modified PNM BA boundaries. **(c)** Initial WALC BA boundaries. **(d)** Modified WALC BA boundaries. **(e)** Initial SWPP sub-BA boundaries. **(f)** Modified SWPP sub-BA boundaries **(g)** Initial PJM sub-BA boundaries. **(h)** Modified PJM sub-BA boundaries.

Figure 5 shows the initial and modified boundaries. Note that BA/sub-BA boundaries represent another source of potential error in the dataset, as there could be other issues in the EIA shapefiles that we did not identify and our modifications ultimately represent a best guess based on inference and publicly available online information. Incorrect boundaries can impact the final county-level load shapes by preventing the correct association of sub-BAs and load loss events (thus leading to improper load loss adjustments or non-adjustments of corresponding load profiles) and producing an incorrect estimation of the hourly consumption patterns of retail sales and direct use for a county, as will be explained in the following section, titled “Estimating hourly retail sales and direct use for each county.”

### Estimating hourly retail sales and direct use for each county

We create an hourly profile of retail sales and direct use for each county and year by determining the BA/sub-BAs that serve the county, estimating each BA/sub-BA’s contribution to the county’s retail sales and direct use for the year, scaling each BA/sub-BA’s load profile for the year according to its estimated contribution, and adding the scaled profiles.

To determine the BA/sub-BAs that serve each county and estimate their contributions to the county’s retail sales and direct use, we follow these steps for each year:

- Determine the spatial intersections between all BA/sub-BAs and U.S. census tracts. Census tracts are small subdivisions of counties, generally containing between 1200 and 8000 people. We obtain census tract boundaries from the U.S. Census Bureau’s 2022 TIGER/Line Tract shapefiles (https://www2.census.gov/geo/tiger/TIGER2022/TRACT/). Note that each census tract belongs entirely to one county.
- Calculate the population of each intersection by multiplying its land area by the population density of the census tract it belongs to. To calculate each census tract’s population density, we obtain census tract populations from the U.S. Census Bureau’s 2020 Decennial Census table (https://data.census.gov/table/DECENNIALDHC2020.P1?q=P1:+TOTAL+POPULATION&g=010XX00US$1400000) and divide the populations by the land areas provided by the census tract shapefiles. We proportionally adjust the calculated populations of the intersections so that, when aggregated to counties, the populations of the intersections sum to the population of the county they belong to.
- Calculate the supply-derived load of each intersection by taking the total load of the intersection’s BA/sub-BA for the year and multiplying it by the intersection’s share of BA/sub-BA population (the total population of all intersections belonging to the BA/sub-BA).
- Calculate the supply-derived load served to each county by each BA/sub-BA by grouping the intersections by the counties and BA/sub-BAs they belong to and taking the sum of the supply-derived loads for each group.
- Calculate each BA/sub-BA’s contribution to each county’s retail sales and direct use for the year by multiplying the county’s retail sales and direct use by the BA/sub-BA’s share of the county’s supply-derived load.

In summary, the population of the intersection between census tract *r* in county *c* and BA/sub-BA *b*, p_*rb*_, is given by

$${{\rm{p}}}_{{rb}}={{\rm{p}}}_{c}\ast {{\rm{V}}}_{{rb}}\ast {d}_{r}/\sum r{\prime} \in {{\rm{R}}}_{c}({{\rm{V}}}_{r{\prime} b}\ast {{\rm{d}}}_{r{\prime} })$$

where p_*c*_ is the population of county *c*, v_*rb*_ is the land area of the intersection between census tract *r* and BA/sub-BA *b*, d_*r*_ is the population density of census tract *r*, and R_*c*_ is the set of all census tracts in county *c*. The supply-derived load of the intersection between census tract *r* and BA/sub-BA *b* in year *t*, m_*rbt*_, is given by

$${{\rm{m}}}_{{rbt}}={{\rm{m}}}_{{bt}}\ast {{\rm{p}}}_{{rb}}/\sum r{\prime} \in {\rm{R}}b({{\rm{p}}}_{r{\prime} b})$$

where m_*bt*_ is the total load of BA/sub-BA *b* in year *t* and R_*b*_ is the set of all census tracts that intersect BA/sub-BA *b*. The supply-derived load served by BA/sub-BA *b* to county *c* in year *t*, m_*cbt*_, is given by

$${{\rm{m}}}_{{cbt}}=\sum r\in {{\rm{R}}}_{c}{{\rm{m}}}_{{rbt}}.$$

**Fig. 6:**
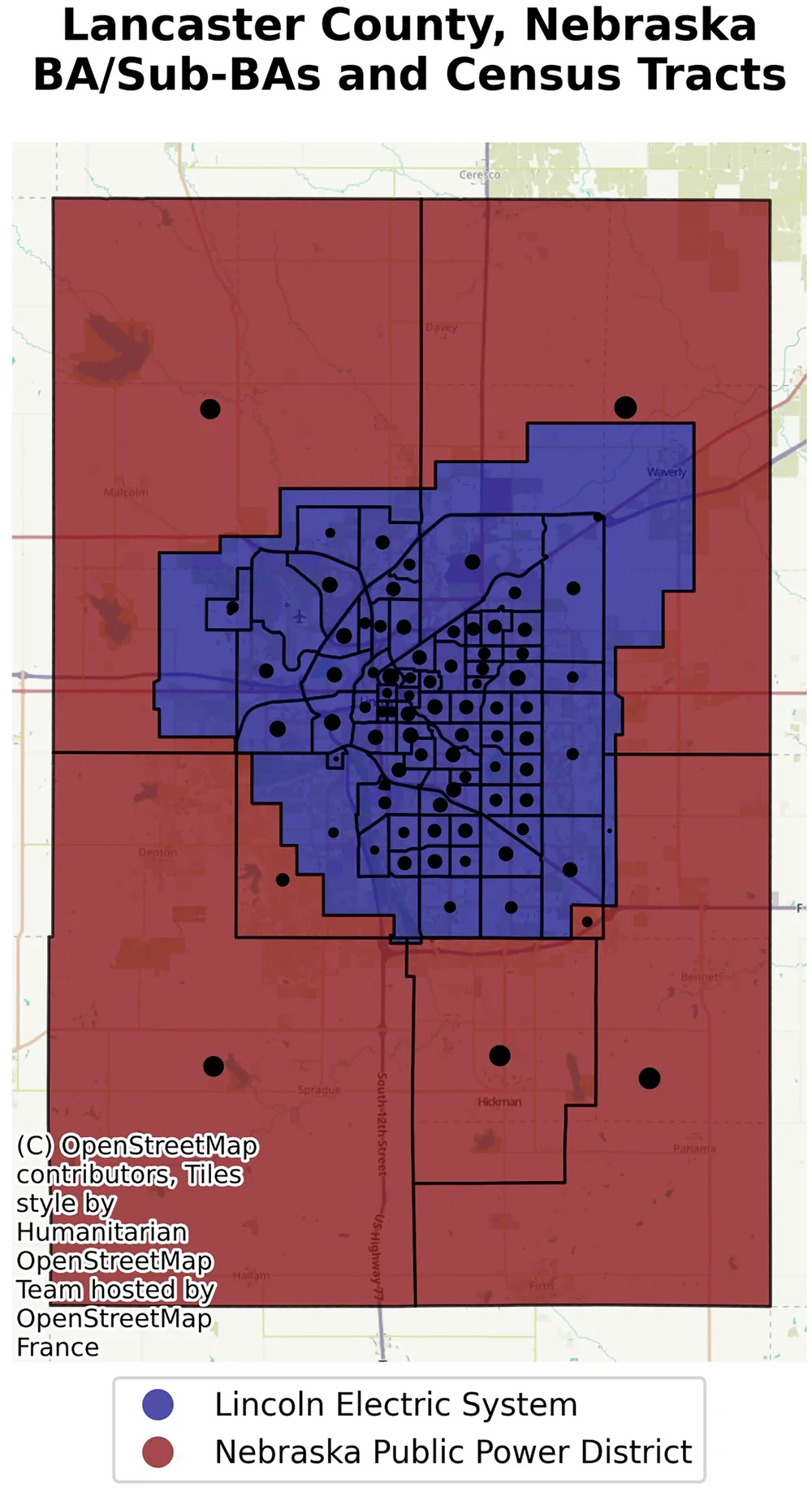
Intersections between BA/sub-BAs serving Lancaster County, Nebraska and the county’s census tracts. The size of the black marker at each intersection’s centroid reflects that intersection’s estimated supply-derived load for 2023 relative to that of the others. Although Nebraska Public Power District (NPPD) covers most of the county’s land area and is a larger system than Lincoln Electric System (LES) in terms of total load, LES serves more population-dense census tracts of the county and a greater number of them. Additionally, LES serves only Lancaster County while NPPD serves many other counties, resulting in a smaller percentage of NPPD’s total load being attributed to Lancaster County. As a result, the estimated proportions of Lancaster County’s 2023 supply-derived load served by LES and NPPD, respectively, are 0.814 and 0.186. Therefore, the contributions of LES and NPPD to Lancaster County’s 2023 retail sales and direct use of 5,559 gigawatt-hours are 4,525 and 1,034 gigawatt-hours, respectively.

BA/sub-BA *b*’s contribution to county *c*’s retail sales and direct use in year *t*, w_*cbt*_, is given by

$${{\rm{w}}}_{{cbt}}={{\rm{m}}}_{{cbt}}/\sum b{\prime} \in {\rm{Bc}}\left({{\rm{m}}}_{{cb}{\prime} t}\right)$$

where B_*c*_ is the set of all BA/sub-BAs that intersect county *c*. Figure 6 shows an example for Lancaster County, Nebraska.

**Fig. 7:**
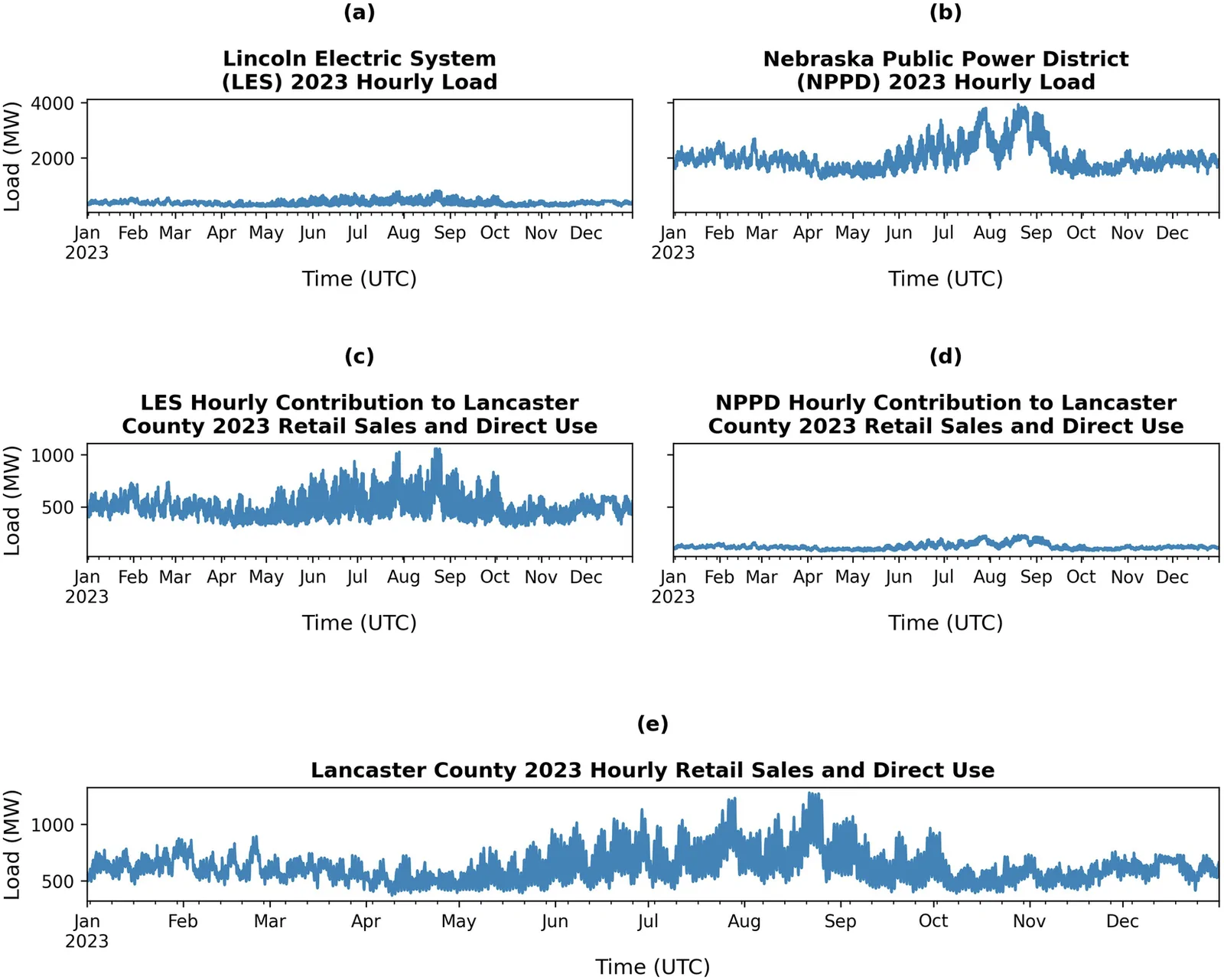
Overview of creating Lancaster County, Nebraska’s 2023 hourly retail sales and direct use profile. (**a)** 2023 hourly load profile for LES. **(b)** 2023 hourly load profile for NPPD. **(c)** 2023 scaled load profile for LES. This is the load profile shown in (**a**), scaled so that its total is equal to 4,525 gigawatt-hours, or LES’s contribution to Lancaster County’s retail sales and direct use for 2023 (see Fig. 6). **(d)** 2023 scaled load profile for NPPD. This is the load profile shown in (**b**), scaled so that its total is equal to 1,034 gigawatt-hours, or NPPD’s contribution to Lancaster County’s retail sales and direct use for 2023 (see Fig. 6). **(e)** 2023 hourly retail sales and direct use profile for Lancaster County. This is the sum of the scaled profiles shown in (**c**) and (**d**).

For each county and year, we scale each BA/sub-BA’s load profiles for that year so that total load matches the BA/sub-BA’s contribution to the county’s retail sales and direct use for that year. We then add the scaled profiles by calculating the sum of the profiles’ values at each hour. Figure 7 shows an example for Lancaster County, Nebraska.

### Collecting county-level DPV capacity factor profiles

We obtain county- and sector-level (residential and commercial) hourly DPV capacity factor profiles from the Renewable Energy Potential Model (reV)^8^. The profiles are modeled using solar resource data from the National Solar Radiation Database (NSRDB)^9^, with each county being represented by the NSRDB point closest to the county’s centroid. Fixed roof mount photovoltaic systems are modeled with a tilt of 16 degrees and an azimuth angle of 180 degrees, which corresponds to a south-facing orientation. Both residential and commercial systems are modeled with a direct-current-to-alternating-current ratio of 1.21, ground cover ratio of 0.4, and direct current system losses of 10.4%. Residential systems are modeled with an inverter efficiency of 96%, and commercial systems are modeled with an inverter efficiency of 98%.

### Estimating annual DPV consumption for each county

We estimate annual county- and sector-level DPV consumption using state- and sector-level estimates of DPV generation and DPV-generated energy that was sold back to the grid, county-level DPV capacity, and county- and sector-level DPV capacity factors. We obtain annual estimates of DPV generation for each state and sector (residential, commercial, and industrial) from the “Estimated Small Scale Generation” columns of the EIA Electric Power Annual (https://www.eia.gov/electricity/annual/) Table 3.21 (“Net generation from solar photovoltaic by state by sector”). We obtain annual reports of energy that was generated by net-metered PV systems and sold back to the grid for each state and sector (residential, commercial, and industrial) from the EIA-861 Net Metering dataset. We obtain annual estimates of installed DPV capacity for each county from the Distributed Generation Market Demand Model (dGen)^10^, used for the 2024 Standard Scenarios^11^.

We calculate annual DPV consumption for each state and sector by subtracting from the state and sector’s total DPV generation the amount of energy sold back to the grid. We estimate annual county- and sector-level DPV consumption by allocating statewide sectoral DPV consumption to counties according to each county’s share of statewide sectoral DPV generation for the given year. The capacity estimates from dGen are not separated by sector, so to calculate county shares of statewide DPV generation, we assume every county of a given state has the same proportions of residential, commercial, and industrial DPV capacity. Under this assumption, each county’s share of statewide DPV generation for a given sector is equal to its share of statewide maximum DPV generation for the sector (DPV generation calculated as if the state’s entire DPV capacity belongs to the given sector). We calculate each county’s annual maximum DPV generation for each sector by taking the sum of the corresponding capacity factors across all hours of the year and multiplying it by the county’s total DPV capacity. We do not have capacity factors for industrial DPV systems, so we use the commercial capacity factors for industrial sector calculations. We then calculate annual county-level shares of DPV generation for each state and sector by dividing each county’s maximum DPV generation by the sum of maximum DPV generation estimates across all counties in the state for the sector and year.

In summary, DPV consumption for state *s* and sector *z* in year *t*, q_*szt*_, is given by

$${{\rm{q}}}_{{szt}}={{\rm{m}}}_{{szt}}-{{\rm{n}}}_{{szt}}$$

where m_*szt*_ and n_*szt*_, respectively, are the DPV generation and DPV-generated energy sold back to the grid for state *s* and sector *z* in year *t*. Maximum DPV generation for county *c* and sector *z* in year *t*, k_*czt*_, is given by

$${{\rm{k}}}_{czt}={{\rm{i}}}_{ct}\ast \sum h\in {{\rm{H}}}_{t}({{\rm{f}}}_{czh})$$

where i_*ct*_ is the total DPV capacity of county *c* in year *t*, f_*czh*_ is the capacity factor value for county *c* and sector *z* at hour *h*, and H_*t*_ is the set of all hours in year *t*. DPV consumption for county *c* and sector *z* in year *t*, q_*czt*_, is given by

$${{\rm{q}}}_{{czt}}={{\rm{q}}}_{{szt}}\ast {{\rm{k}}}_{{czt}}/\sum c{\prime} \in {{\rm{C}}}_{s}({{\rm{k}}}_{c{\prime} {zt}})$$

where C_*s*_ is the set of all counties in state *s*.

### Estimating hourly DPV consumption for each county

**Fig. 8:**
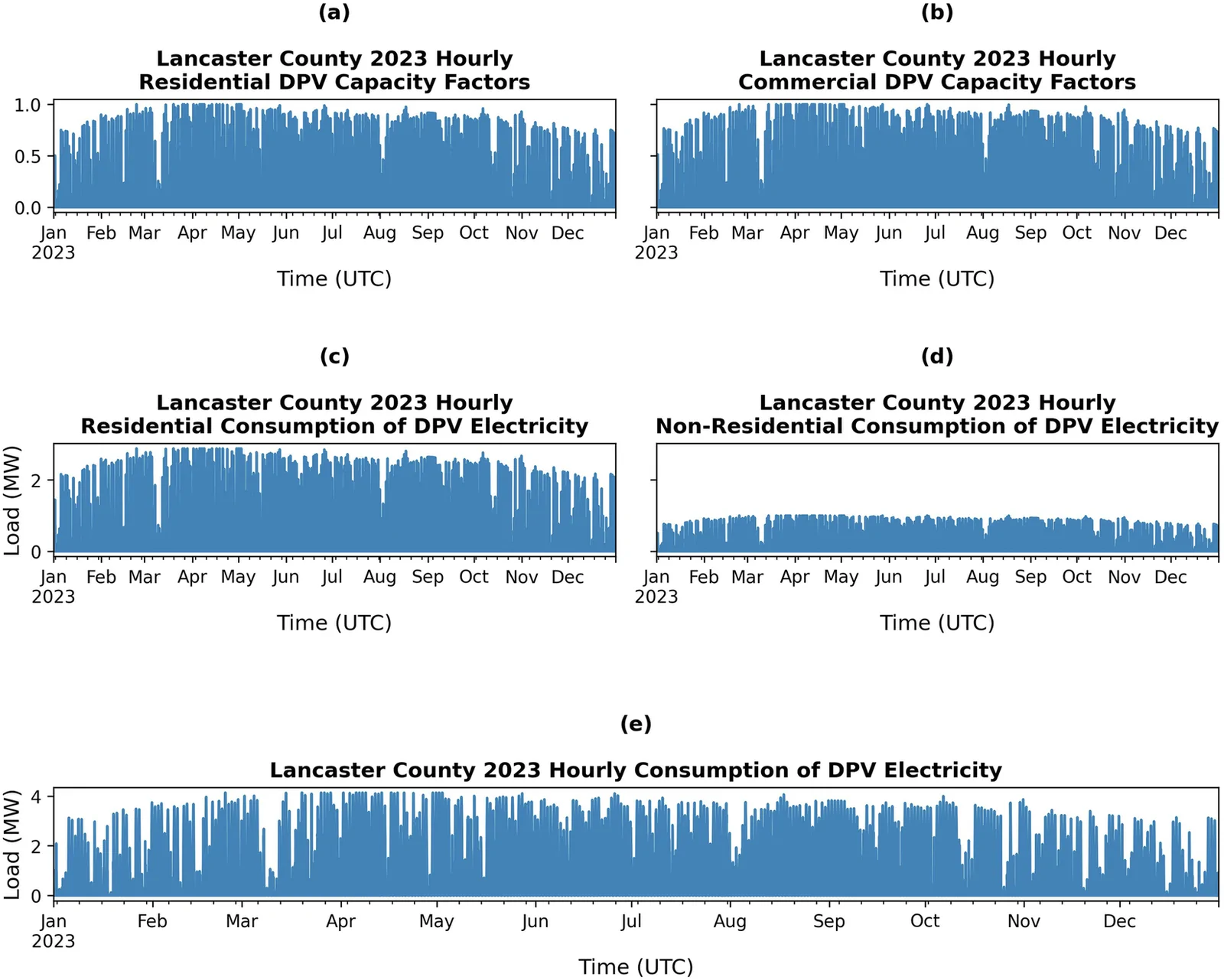
Overview of creating Lancaster County, Nebraska’s 2023 hourly DPV consumption profile. (**a)** 2023 residential DPV capacity factors. **(b)** 2023 commercial DPV capacity factors. **(c)** 2023 hourly residential DPV consumption. This is the capacity factor profile shown in (**a**), scaled so that its total is equal to 5055 megawatt-hours, or Lancaster County’s residential DPV consumption for 2023. **(d)** 2023 hourly non-residential DPV consumption. This is the capacity factor profile shown in (**b**), scaled so that its total is equal to 2257 megawatt-hours, or Lancaster County’s commercial and industrial DPV consumption for 2023. **(e)** 2023 hourly DPV consumption for Lancaster County. This is the sum of the scaled profiles shown in (**c**) and (**d**).

We create an hourly profile of DPV consumption for each county and year by scaling the county’s residential and commercial DPV capacity factor profiles for that year so that their respective totals match the county’s residential and non-residential DPV consumption for the year and then adding the scaled profiles. Figure 8 shows an example for Lancaster County, Nebraska.

### Creating county-level load profiles

**Fig. 9:**
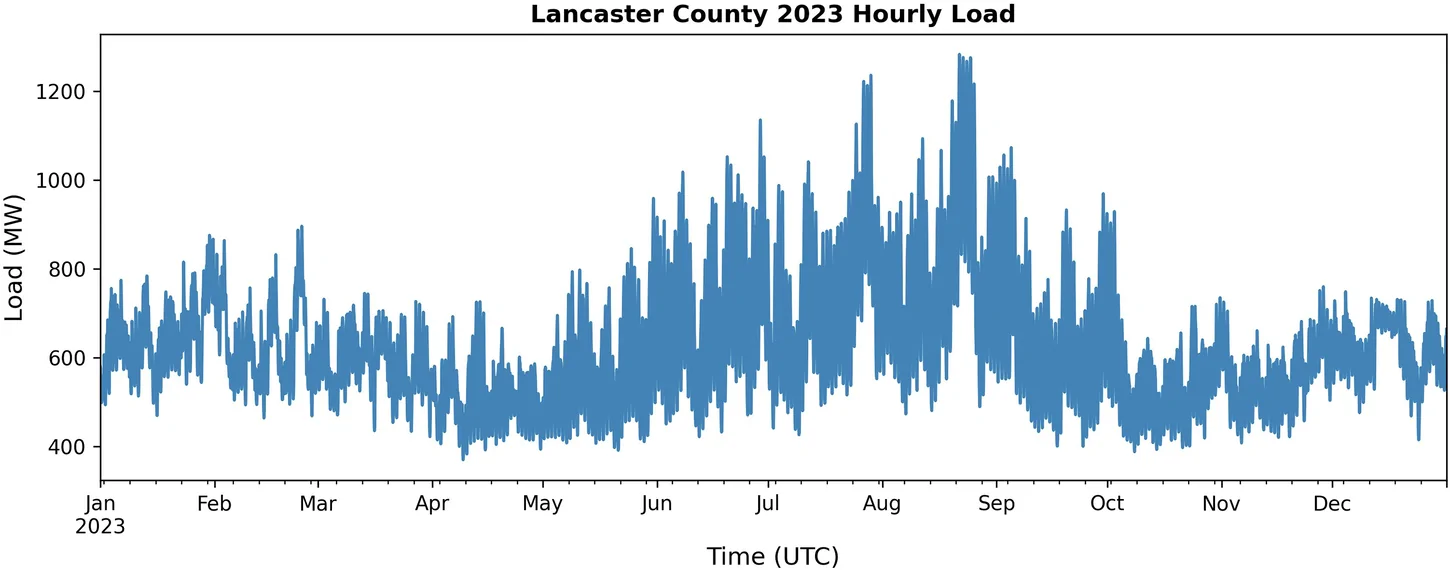
Lancaster County, Nebraska’s 2023 hourly load profile. This is the sum of the hourly retail sales and direct use profile shown in Fig. 7e and the hourly DPV consumption profile shown in Fig. 8e.

To create an hourly load profile for each county and year, we add the hourly profile of retail sales and direct use and the hourly profile of DPV consumption for that county and year. Figure 9 shows the 2023 hourly load profile for Lancaster County, Nebraska.

**Fig. 10:**
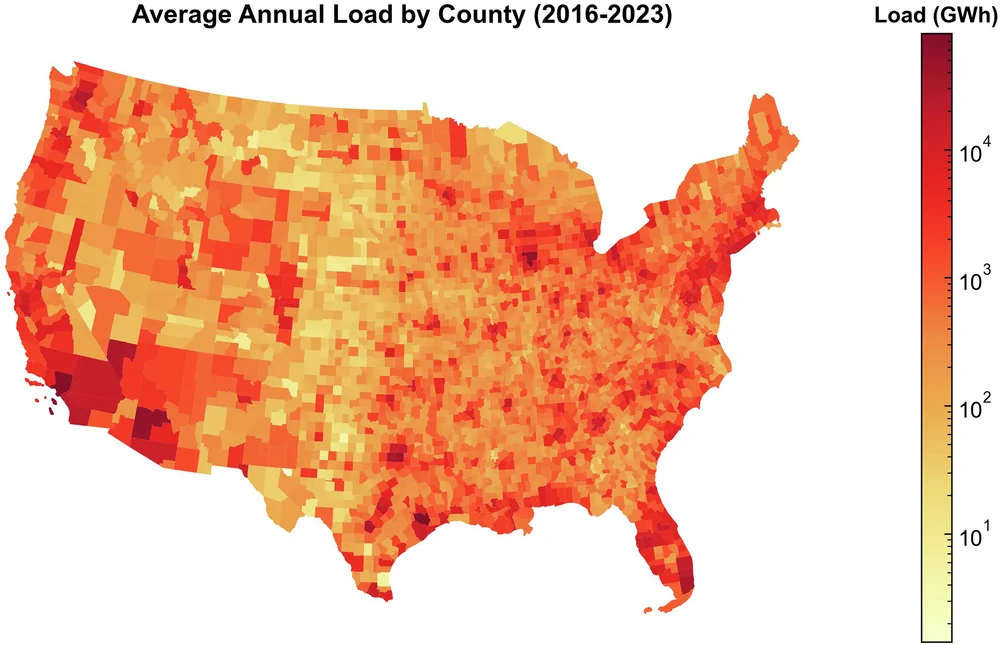
Average annual load for each county in the contiguous U.S. (2016–2023).

Using the hourly load estimates created by this process, Fig. 10 shows the average annual load for each county for 2016 through 2023.

## Data Records

The 2016–2023 county-level hourly load profiles created by this process are contained in the “Historic Load Hourly 2016-2023.h5” file at https://data.openei.org/submissions/8562^14^. Each column contains the hourly load in megawatts for one county, with the column name being the county’s Federal Information Processing Standard (FIPS) code prepended with a “p” (e.g., “p36041” represents Hamilton County, New York). The data are published in UTC and use the hour ending time reporting convention.

## Technical Validation

### Ensuring consistency between load sources

**Fig. 11:**
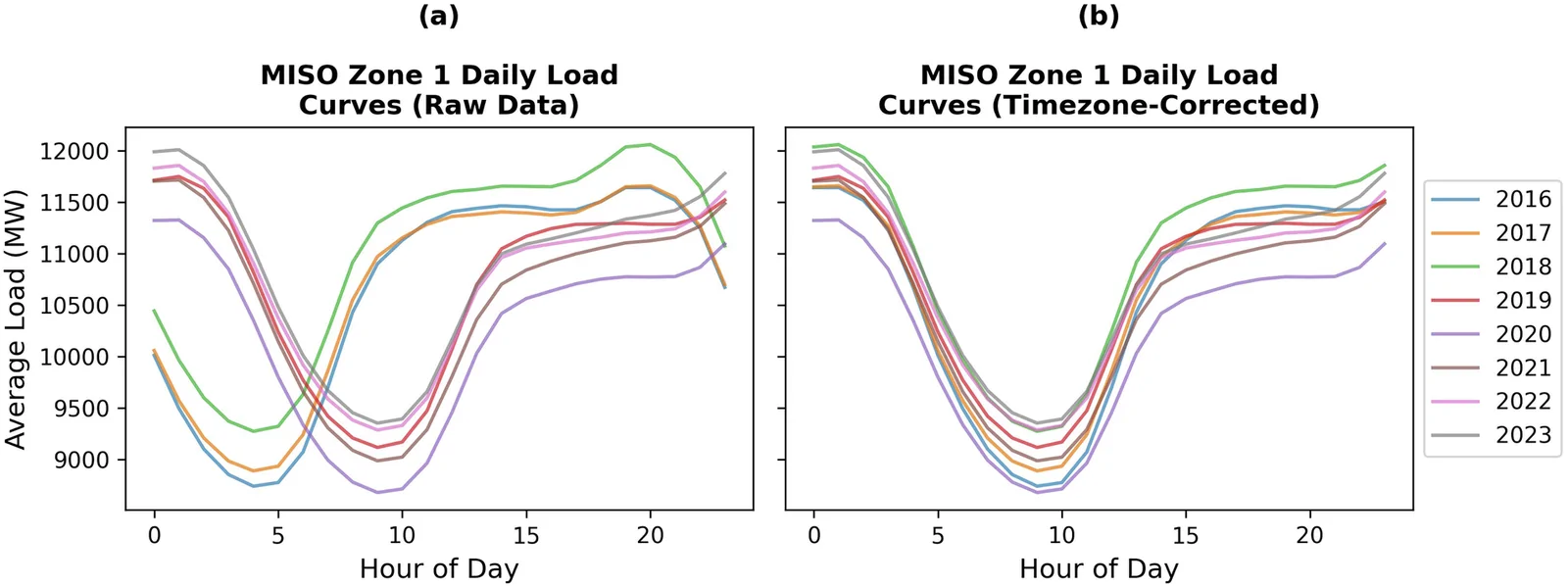
Annual daily load curves for MISO Zone 1 (**a**) as collected from load sources and (**b**) after timezone correction. Daily load curves represent the average load for each hour of day in the given year. Because MISO publishes load data in Eastern Standard Time (EST) and EIA-930 publishes data in UTC, Zone 1’s pre-2019 load initially has a five-hour time lag compared to the 2019-2023 load. After modification, the load curves are consistent across all years.

As discussed in “Methods—Collecting BA and sub-BA load and load forecast profiles,” EIA-930 publishes load in UTC using the hour ending time reporting convention, and we modify load data from other sources for consistency with EIA-930. Specifically, in cases where external load data is published in a non-UTC timezone, we shift each load value’s timestamp forward according to the timezone’s UTC offset (e.g., the timestamps for load published in Eastern Standard Time are shifted forward by five hours). In cases where external load data is published using the hour beginning time reporting convention, we shift each timestamp forward by one hour. To validate consistency across load sources after these modifications, we plotted the daily load curves (average load for each hour of day in a given year) across years for each sub-BA. These plots also allowed us to confirm that load magnitudes for a given sub-BA are generally in line with each other across different data sources. An example for MISO’s Zone 1 sub-BA is shown in Fig. 11.

### Evaluating annual consumption estimates and estimation methods

Our overall method for estimating annual electricity consumption for each county is outlined in “Methods—Estimating annual retail sales for each county,” “Methods—Calculating annual direct use for each county,” and “Methods—Estimating annual DPV consumption for each county.” We assess the accuracy of this method by comparing our estimates of 2023 county-level consumption to those published by California and New Jersey. For California, we take consumption values from the California Energy Commission (CEC) “AGG_CONSUMPTION_ELEC_COUNTY” table (https://www.energy.ca.gov/files/energy-consumption-data-files), which the CEC compiles using county-level sales and self-generation data reported by utilities and other entities. For New Jersey, we take consumption values from the Sustainable Jersey “Aggregated Community-Scale Utility Energy Data” dataset (https://www.sustainablejersey.com/resources/data-center/sustainable-jersey-data-resources/), which contains county-level sales reported by utilities.

**Fig. 12:**
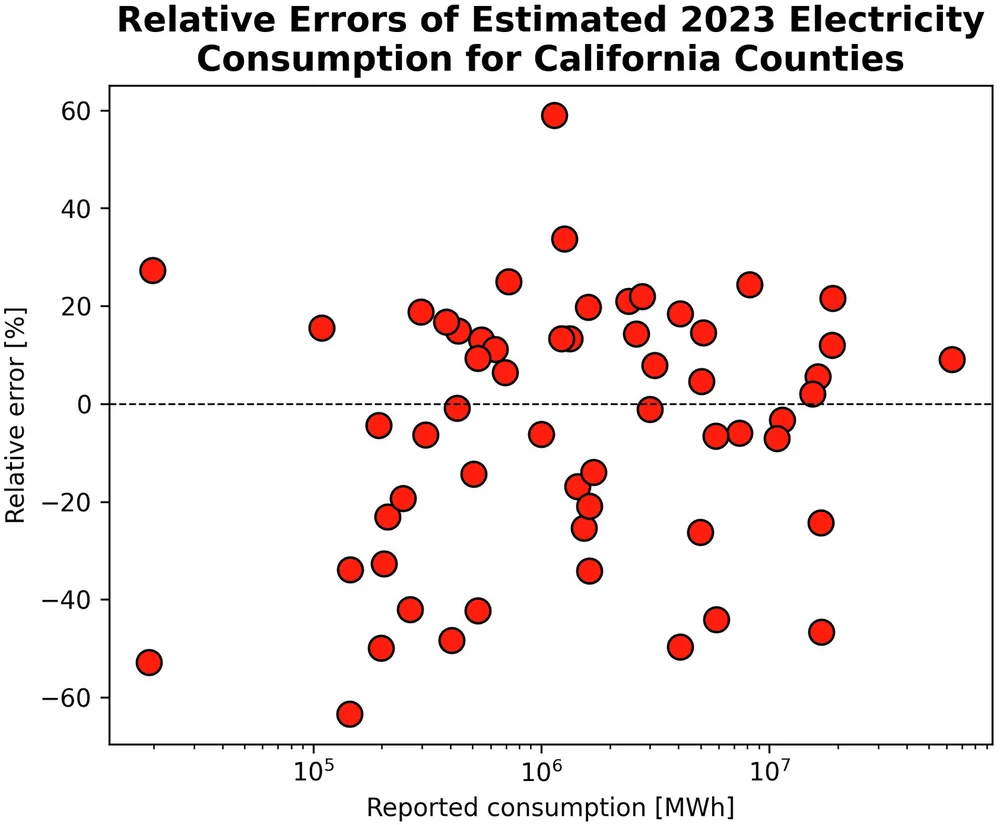
Relative errors of estimated 2023 electricity consumption for California counties.

**Fig. 13:**
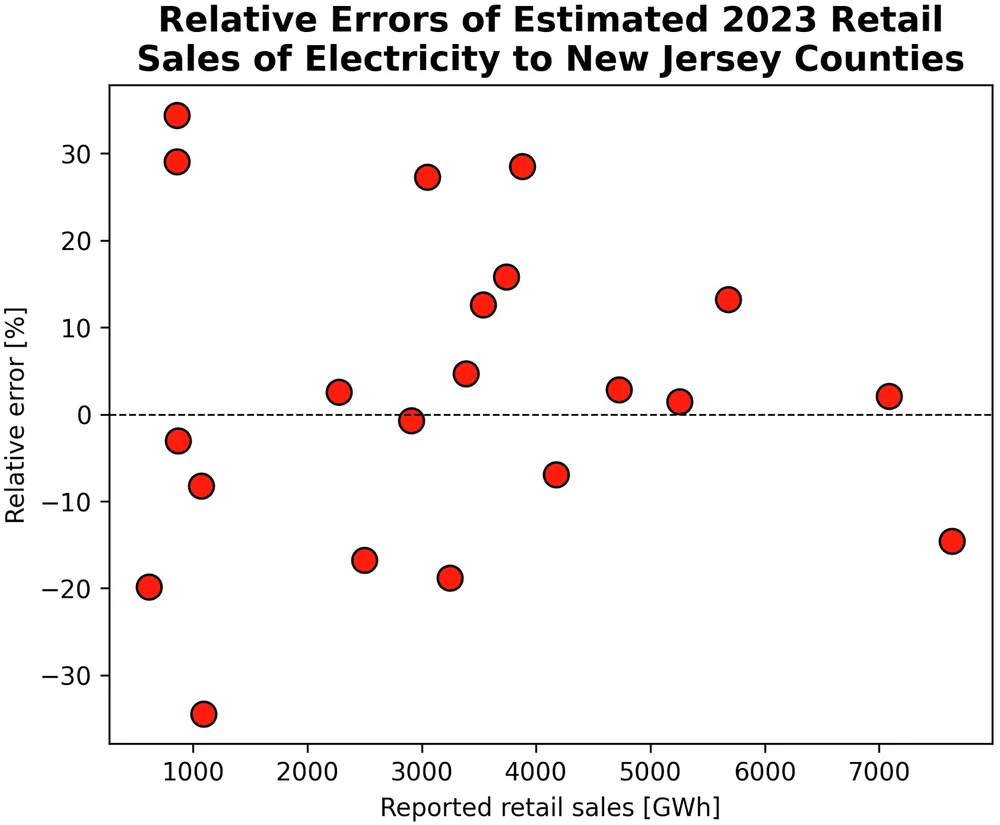
Relative errors of estimated 2023 retail sales of electricity to New Jersey counties.

Because the CEC incorporates the same components as we do to derive total county-level electricity consumption (retail sales and on-site consumption of self-generated electricity), we compare our total consumption estimates to their published values. Sustainable Jersey’s dataset only reflects retail sales, so we compare only our retail sales estimates to their published values. We calculate the relative error of consumption for each county by taking the difference between our estimate and the reported value, dividing the difference by the reported value, and multiplying the result by 100. We calculate the absolute relative error by taking the absolute value of the relative error. Figures 12 and 13 show the relative errors for each county in California and New Jersey respectively, plotted against their reported consumption.

For California, the median, mean, and maximum absolute relative error respectively are 18.4%, 21.7%, and 63.3%. There is also a slight difference between our estimate for California’s statewide consumption in 2023 (275.1 terrawatt-hours) and the CEC’s statewide consumption figure (276.7 terrawatt-hours), suggesting that, irrespective of the allocation of statewide consumption, some county-level differences also stem from differences in the raw consumption data collected or estimated by the EIA and CEC. For New Jersey, the median, mean, and maximum absolute relative error respectively are 13.2%, 14.2%, and 34.4%. In this case, there is also a difference between our statewide retail sales (70.2 terrawatt-hours) and those reported by Sustainable Jersey (68.4 terrawatt-hours) due to some smaller utilities not reporting to Sustainable Jersey and data for some areas being suppressed by reporting utilities for customer privacy reasons.

Despite the statewide differences in both cases, as discussed in “Methods—Estimating annual retail sales for each county” the county-level errors are most likely attributable to the population-based allocation of statewide retail sales to counties, which is inherently imprecise due to the lack of consideration for other factors that might influence a county’s actual share of statewide sales (e.g., commercial and industrial activity). While developing this dataset, we explored a variety of other methods for estimating county-level retail sales. We experimented only with retail sales because they constitute the vast majority (95%) of nationwide electricity consumption and allocating them based on population is the largest source of uncertainty in the annual county-level consumption estimates. The alternative methods are outlined below.

- Sales allocation by Gross Domestic Product (GDP): We allocate statewide retail sales to counties according to each county’s share of state GDP. We obtain 2023 real GDP by county from the U.S. Bureau of Economic Analysis (BEA)’s “CAGDP1 County and MSA gross domestic product (GDP) summary” table^12^.
- Sales allocation by population and sectoral GDP: We split up statewide retail sales by sector (residential, commercial, industrial, and transportation), as specified in EIA-861. We allocate residential sales to counties according to each county’s share of state population. We allocate industrial sales to counties according to each county’s share of state GDP of the construction, manufacturing, mining, quarrying, oil/gas extraction, agriculture, forestry, fishing, and hunting industries, which we obtain from the BEA’s “CAGDP9 Real GDP by industry in chained dollars” table^13^. Note the EIA-861 form specifies manufacturing, construction, mining, agriculture, fishing, and forestry establishments as components of the industrial sector, and we include other industries like oil/gas extraction and hunting because the BEA groups them with the industries specified by EIA-861. We treat GDP values that are hidden by the BEA to protect confidential information (represented by a “(D)” in the data table) as 0 in our calculations. We allocate commercial and transportation sales to counties according to each county’s share of remaining state GDP (the portion of state GDP not considered in the allocation of industrial sales).
- Sales allocation by estimated supply-derived load: Using the estimates of supply-derived load served by each BA/sub-BA to each county calculated in “Methods–Estimating hourly retail sales and direct use for each county,” we calculate total supply-derived load for each county and state. We allocate statewide retail sales to counties according to each county’s share of state supply-derived load.
- Sales allocation by population using utility service areas: We obtain the list of counties served by each utility from the EIA-861 Service Territories dataset. For each utility and state, we allocate retail sales to counties according to each county’s share of the total population of counties belonging to the state and served by the utility. We allocate remaining statewide retail sales (e.g., retail sales made directly by power plants) to counties according to each county’s share of the statewide sales made by utilities.

**Fig. 14:**
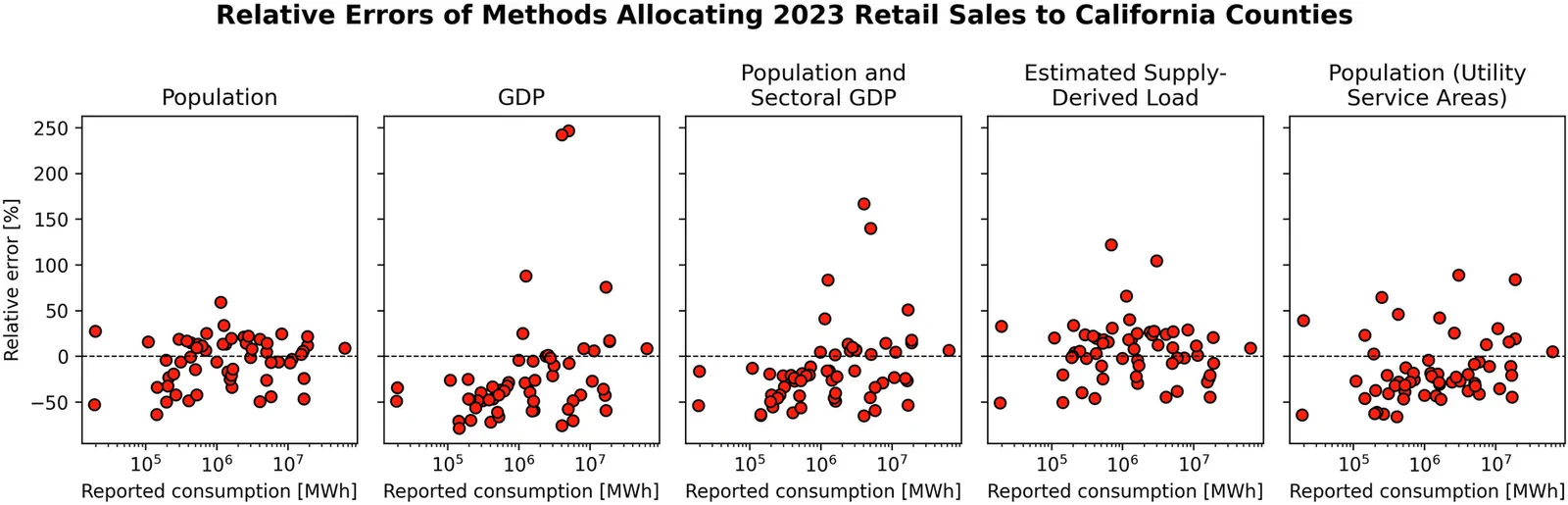
Relative errors of estimated 2023 electricity consumption for California counties using different methods for allocating retail sales to counties.

**Fig. 15:**
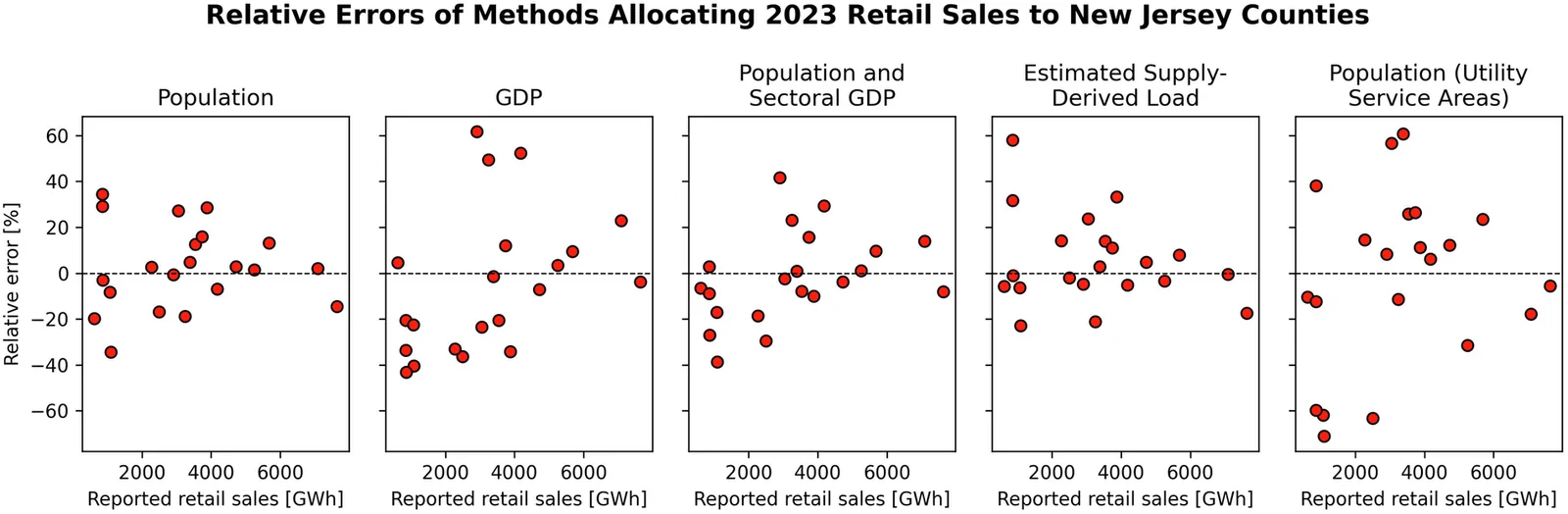
Relative errors of estimated 2023 retail sales of electricity to New Jersey counties using different methods for allocating retail sales to counties.

We calculate the relative errors and absolute relative errors of consumption for each county using each method. Figures 14 and 15 show the relative errors for each county in California and New Jersey respectively, plotted against their reported consumption and titled by the method used. Table 3 shows the median, mean, and maximum absolute relative errors for each method. For California, allocating retail sales by population produces the lowest median, mean, and maximum absolute relative error. For New Jersey, allocating by estimated supply-derived load produces the lowest median and mean absolute relative error, and allocating by population produces the lowest maximum absolute relative error.

**Table 3:** Summary statistics of absolute relative errors of estimated 2023 electricity consumption for California counties and 2023 retail sales of electricity to New Jersey counties using different methods for allocating retail sales to counties.

State	Method	Median error (%)	Mean error (%)	Maximum error (%)
California	Population	18.4	21.7	63.3
California	GDP	41.0	46.0	246.5
California	Population and Sectoral GDP	26.7	34.7	166.6
California	Estimated Supply-Derived Load	21.2	24.3	126.7
California	Population (Utility Service Areas)	30.0	32.6	89.9
New Jersey	Population	13.2	14.2	34.4
New Jersey	GDP	23.0	25.5	61.6
New Jersey	Population and Sectoral GDP	10.0	15.1	41.7
New Jersey	Estimated Supply-Derived Load	7.9	13.9	58.1
New Jersey	Population (Utility Service Areas)	23.5	29.9	71.1

### Evaluating hourly load patterns

In the absence of actual county-level load profiles to assess our county-level profiles against, we evaluate our hourly load patterns by creating state-level hourly profiles reflecting California’s 2023 retail sales and direct use and total electricity consumption and comparing them to California’s 2023 hourly supply-derived load, as reported in EIA-930. EIA creates the hourly supply-derived load profile for the state by aggregating the BA-level data they collect and then imputing the aggregated data.

**Fig. 16:**
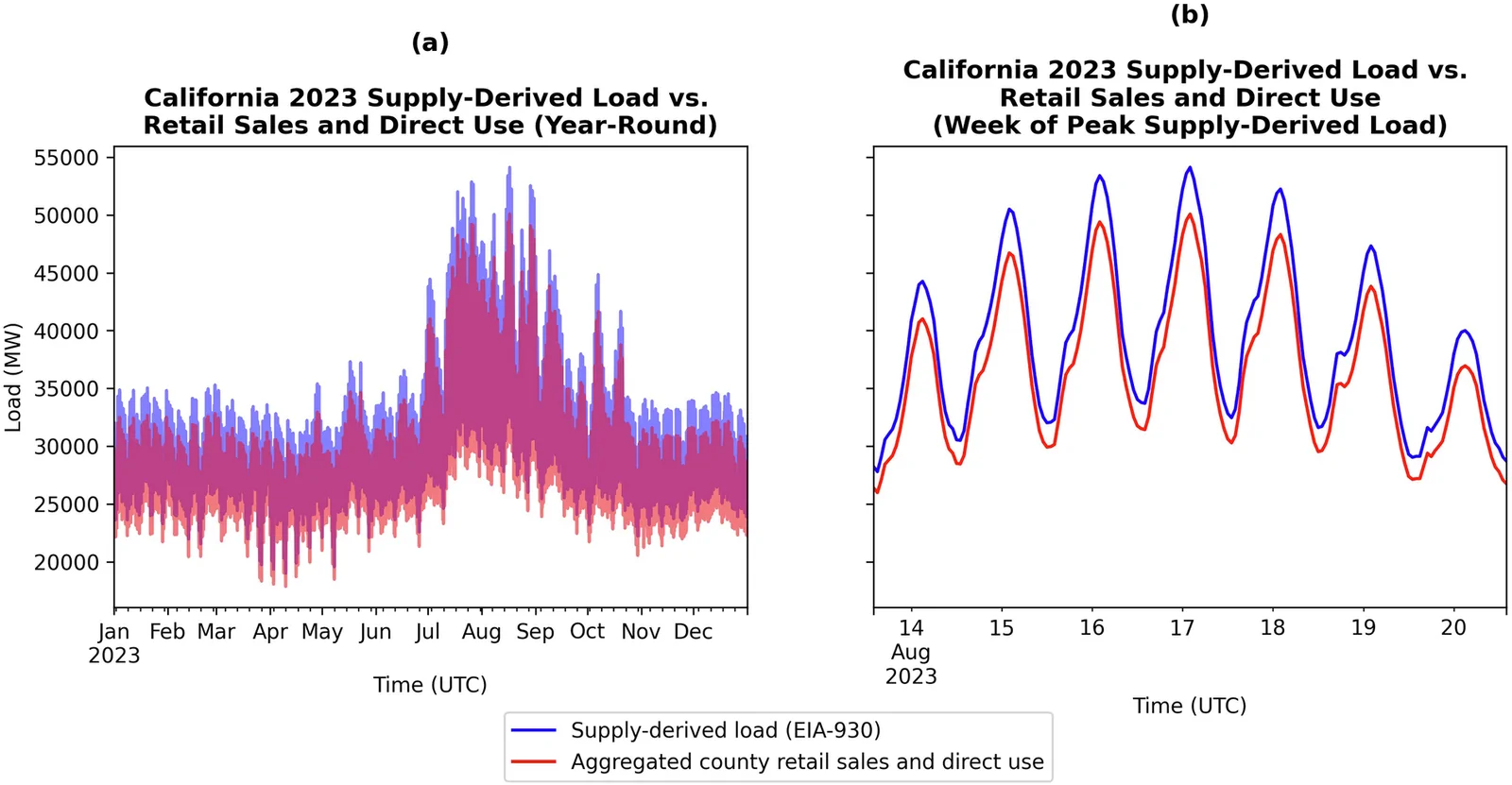
California’s 2023 hourly supply-derived load and aggregated county-level retail sales and direct use (**a**) for the year and (**b**) for the week containing the hour of peak supply-derived load for the year.

We create a 2023 retail sales and direct use profile for California by adding the county-level 2023 retail sales and direct use profiles for California’s counties, as created in “Methods–Estimating hourly retail sales and direct use.” Since our methods assume retail sales, direct use, and supply-derived load have the same hourly load patterns, we expect the hourly patterns of the retail sales and direct use profile and the supply-derived load profile for California to be nearly identical. However, we also anticipate some differences, since the supply-derived load profile does not include adjustments for load loss and the EIA uses its own imputation method to correct anomalies in the aggregated load profiles published in EIA-930, as opposed to the MICE technique we use. Figure 16 shows the supply-derived load profile and the retail sales and direct use profile for the entire year of 2023 and for the week containing the hour of California’s peak supply-derived load for 2023. The Pearson correlation coefficient between the hourly values of the profiles is 0.997, indicating a very strong positive linear correlation between them and suggesting the profiles’ hourly patterns are nearly identical.

**Fig. 17:**
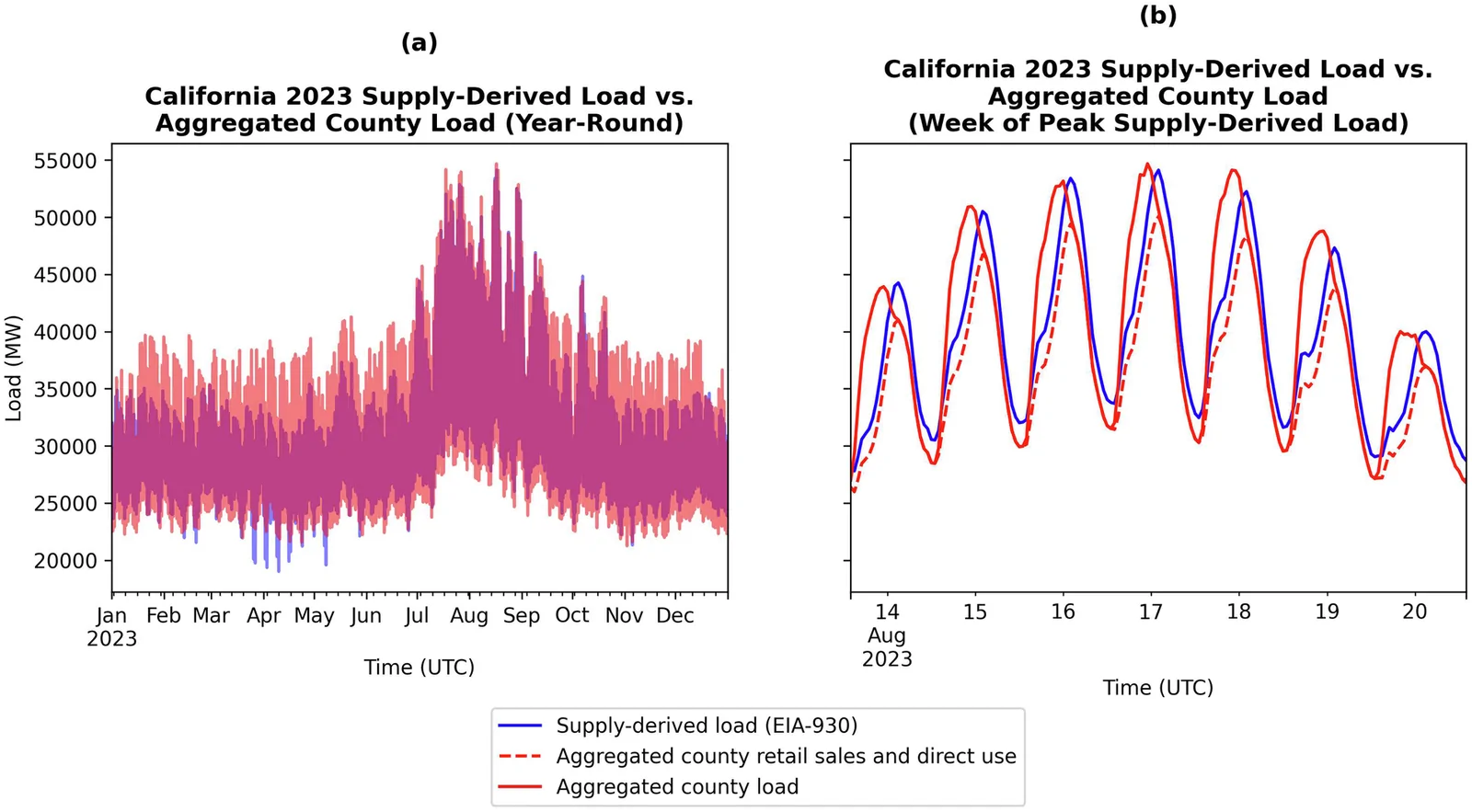
California’s 2023 hourly supply-derived load and aggregated county-level load (**a**) for the year and (**b**) for the week containing the hour of peak supply-derived load for the year. The aggregated county-level retail sales and direct use profile is also included in (**b**) to illustrate the impact of including DPV consumption on the hourly load patterns. In addition to an overall increase in load, a larger percentage of load occurs earlier in the day.

We create a 2023 load profile for California by adding our 2023 county-level load profiles for California’s counties. In this case, we expect the hourly patterns of our load profile to deviate non-trivially from those of the supply-derived load profile due to the inclusion of DPV consumption. Specifically, DPV consumption largely occurs during daytime hours while other types of load typically peak in the evening. Therefore, its inclusion results in a greater share of daily load occurring earlier in the day in our load profile. Figure 17 shows the supply-derived load profile and our load profile for the entire year of 2023 and for the week containing the hour of California’s peak supply-derived load for 2023. The Pearson correlation coefficient between the hourly values of the profiles is 0.565, indicating a moderate positive linear correlation between them and reflecting the weakened similarity in their hourly patterns relative to the similarity between the retail sales and direct use and supply-derived load profiles.

## Data Availability

The 2016–2023 county-level hourly load profiles created by this process are contained in the “Historic Load Hourly 2016-2023.h5” file at https://doi.org/10.25984/3366592^14^.

## Footnotes

1 ERCOT weather zones (https://www.ercot.com/files/assets/2023/04/26/Weather.jpg)

## Code availability

The code used to develop this dataset is available at https://github.com/NatLabRockies/HistoricalLoadData.

## References

[1] Draxl, C., Clifton, A., Hodge, B.-M. & McCaa, J. The Wind Integration National Dataset (WIND) Toolkit. *Applied Energy* **151**, 355–366 (2015).

[2] Pfenninger, S. Dealing with multiple decades of hourly wind and PV time series in energy models: A comparison of methods to reduce time resolution and the planning implications of inter-annual variability. *Applied Energy* **197**, 1–13 (2017).

[3] Milligan, M. *et al*. Capacity value assessments of wind power. *Wiley Interdisciplinary Reviews: Energy and Environment* **6**, e226 (2017).

[4] Shaner, M. R., Davis, S. J., Lewis, N. S. & Caldeira, K. Geophysical constraints on the reliability of solar and wind power in the United States. *Energy and Environmental Science* **11**, 914–925 (2018).

[5] Frysztacki, M. M., Hörsch, J., Hagenmeyer, V. & Brown, T. The strong effect of network resolution on electricity system models with high shares of wind and solar. *Applied Energy* **291**, 116726 (2021).

[6] Brinkerink, M., Mayfield, E. & Deane, P. The role of spatial resolution in global electricity systems modelling. *Energy Strategy Reviews* **53**, 101370 (2024).

[7] Ruggles, T. H., Farnham, D. J., Tong, D. & Caldeira, K. Developing reliable hourly electricity demand data through screening and imputation. *Sci Data* **7**, 155 (2020). 10.1038/s41597-020-0483-x PMC7250876 32457368

[8] Maclaurin, G., Grue, N., Lopez, A. & Heimiller, D. *The Renewable Energy Potential (reV) Model: A Geospatial Platform for Technical Potential and Supply Curve Modeling*. https://www.nrel.gov/docs/fy19osti/73067.pdf (2019).

[9] Sengupta, M. *et al*. The National Solar Radiation Data Base (NSRDB). *Renewable and Sustainable Energy Reviews* **89**, 51–60 (2018).

[10] Sigrin, B., Gleason, M., Preus, R., Baring-Gould, I. & Margolis, R. *The Distributed Generation Market Demand Model (dGen): Documentation*. http://www.nrel.gov/docs/fy16osti/65231.pdf (2016).

[11] Gagnon, P. J. *et al*. *2024 Standard Scenarios Report: A U.S. Electricity Sector Outlook*. https://www.nrel.gov/docs/fy25osti/92256.pdf (2024).

[12] U.S. Bureau of Economic Analysis, CAGDP1 County and MSA gross domestic product (GDP) summary (accessed Thursday, October 23, 2025).

[13] U.S. Bureau of Economic Analysis, CAGDP9 Real GDP by county and metropolitan area (accessed Thursday, October 23, 2025).

[14] Obika, K., Cole, W. & Rivers, M. Hourly Electricity Demand Profiles for Each County in the Contiguous United States. [Data set]. Open Energy Data Initiative (OEDI). National Laboratory of the Rockies (NLR). 10.25984/3366592 (2025).

